# Supplementary material for: Ketohexokinase-A acts as a nuclear protein kinase that mediates fructose-induced metastasis in breast cancer
Source: Nat Commun. 2020 Oct 28;11:5436. doi: 10.1038/s41467-020-19263-1 (PMC7595112; doi:10.1038/s41467-020-19263-1)
Supplement: Supplementary file 1 — Supplementary Information [file 41467_2020_19263_MOESM1_ESM.pdf]

Supplementary Information

Ketohexokinase-A acts as a nuclear protein kinase that mediates fructose-induced metastasis in breast cancer

Kim *et al.*

Supplementary Figure 1. Fructose enhances the invasion potential of various cancer cells

Supplementary Figure 2. KHK-A and KHK-C expression level analysis from TCGA dataset

Supplementary Figure 3. Fructose enhances the invasion ability in KHK-A expressing cancer cells

Supplementary Figure 4. Effect of ALDOB, ALOX12 or PRPS1 on invasion of fructose treated cancer cell

Supplementary Figure 5. KHK-A promotes the fructose-induced metastasis in *in vivo*

Supplementary Figure 6. KHK-C shows no effect on the fructose-induced metastasis

Supplementary Figure 7. Fructose promotes KHK-A binding to LRRC59 and YWHAH

Supplementary Figure 8. Translocalization of KHK-A to the nucleus under fructose stimuli

Supplementary Figure 9. KHK-A functions as a nuclear protein kinase

Supplementary Figure 10. Validation of kinase function of KHK-A

Supplementary Figure 11. YWHAH-pSer25 promotes breast cancer metastasis in fructose-fed mice.

Supplementary Figure 12. YWHAH -pSer25 represses transcriptional activity of CDH1

Supplementary Figure 13. The YWHAH- pSer25 is associated with breast cancer metastasis.

Supplementary Figure 14. Uncropped western blots used in the study

Supplementary Figure 15. MS/MS spectra and fragmented ions in *in vitro* PTM analysis

Supplementary Figure 16. MS/MS spectra and fragmented ions in *in vivo* PTM analysis

Supplementary Table 1. Nucleotide sequences of siRNAs

Supplementary Table 2. Nucleotide sequences of shRNAs

Supplementary Table 3. Primers used in real-time quantitative PCR

Supplementary Table 4. Clinical information on breast cancer patients

Supplementary Information

Ketohexokinase-A acts as a nuclear protein kinase that mediates fructose-induced metastasis in breast cancer

Kim *et al.*

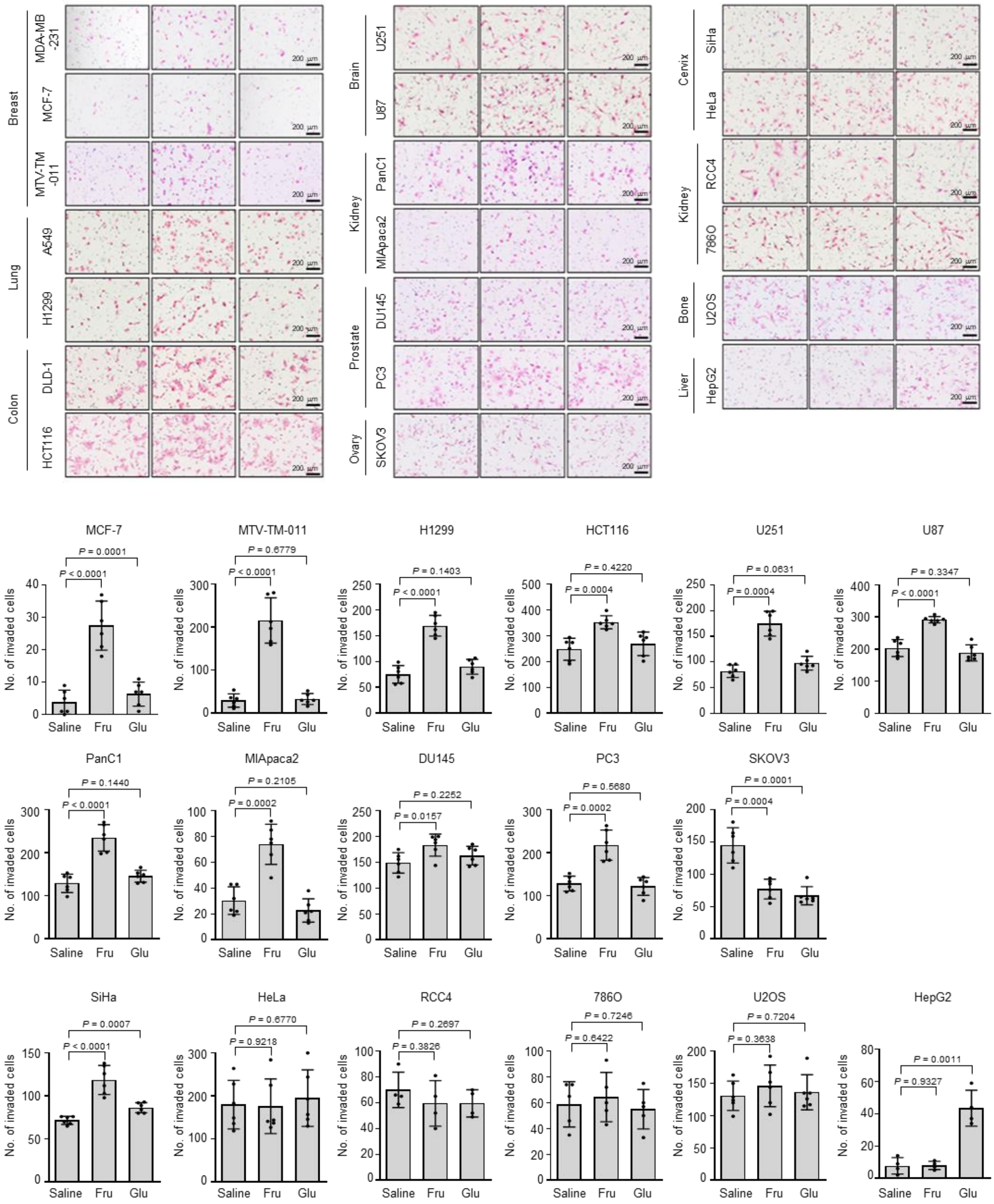

Supplementary Figure 1. Fructose enhances the invasion potential of various cancer cells. Indicated cell lines were incubated with 5 mM fructose or additional 5 mM glucose for 48 hours. Cells were subjected to Matrigel-coated transwell invasion assay (means ± S.D. from $n = 4$ independent experiment for RCC4 and HepG2; means ± S.D. from $n = 5$ independent experiment for 786O; and means ± S.D. from $n = 6$ independent experiment for rest of the cell lines). Significance was calculated by an unpaired, two-sided, Student's t-test, and P-values are represented in each panel. Representative photographs of in vasion assays from one out of three experiments.

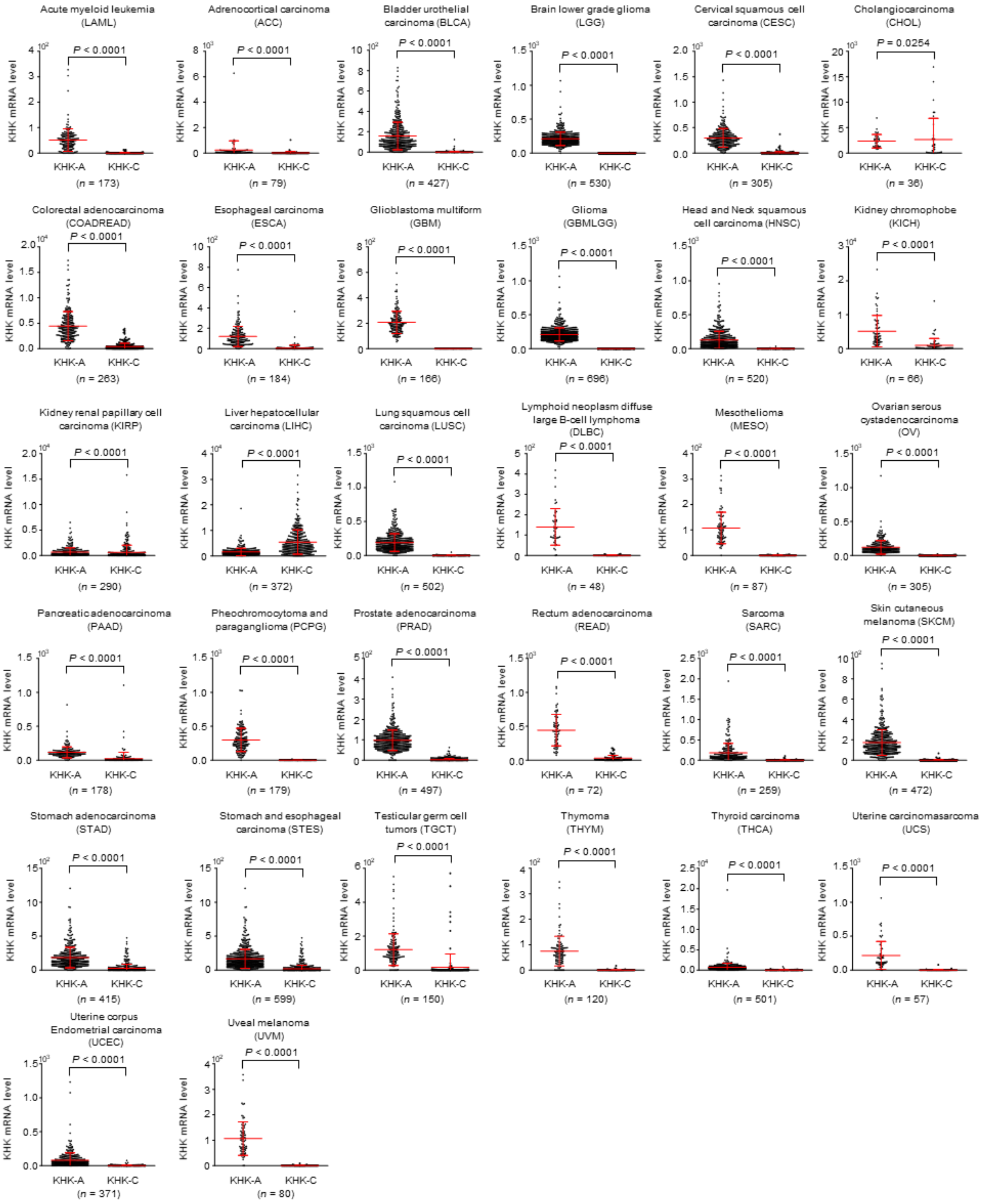

Supplementary Figure 2. KHK-A and KHK-C expression level analysis from TCGA dataset. The mRNA levels of KHK-A and KHK-C in various cancers on TCGA data sets. Data represented as mean s ± S.D. from the number of samples derived from independent cancer patients were provided by TCGA database are shown in each panel. To evaluate significance, two-sided Mann-Whitney *U* test was used.

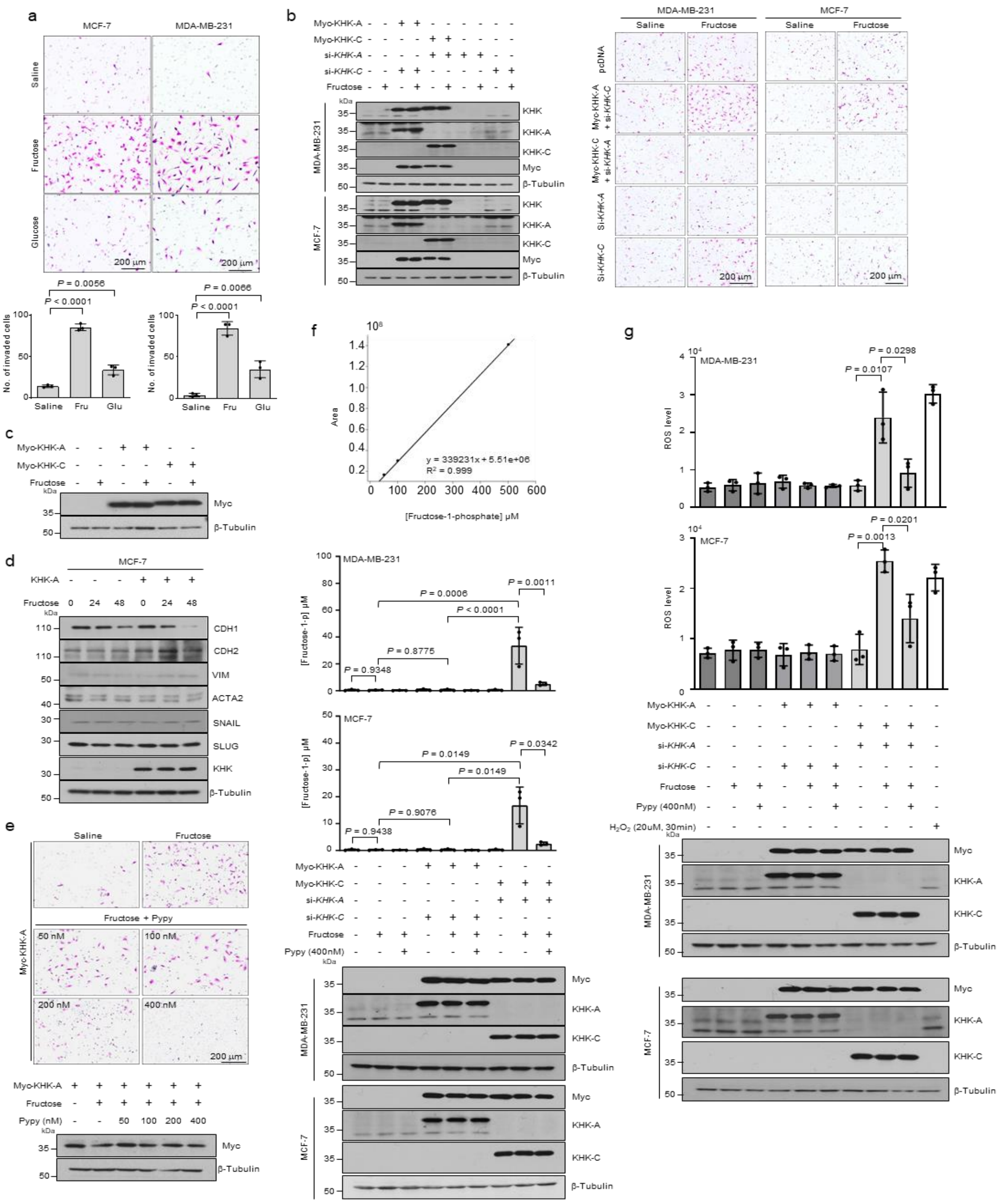

Supplementary Figure 3. Fructose enhances the invasion ability in KHK-A expressing cancer cells. a. MDA-MB-231 and MCF-7 cells incubated with 5 mM fructose or 5 mM glucose in glucose-free medium for 24 hours, and subjected to invasion assay (means ± S.D. from *n* = 3 independent experiments). Significance was calculated by an unpaired, two-sided Student's *t*-test. Representative photographs of Matrigel-coated transwell invasion assays are presented at the top panel. b.

Representative pictures of cell invasion (Right) and Western blots (Left), related to Figure 1d. c. Western blots to verify the transfection efficiency of Myc-KHK-A or Myc-KHK-C in MDA-MB-231 cells, related to Figure 1e. d. MCF-7 stable cells expressing KHK-A were incubated with 5 mM fructose (or saline) for 24 or 48 hours, and subjected to Western blotting for EMT markers. e. MDA-MB-231 cells, which had been transfected with Myc-KHK-A, were treated with 5 mM fructose and/or Pyrimidinopyrimidine for 48 hours, and subjected to invasion assay. Representative photographs of invasion assays performed in the main figure 1h. The transfection efficiency showed in the lower panel. f. MDA-MB-231 and MCF-7 cells, which had been transfected with 1μg of Myc-tagged plasmid or 60 nM si-RNA, were treated with 5 mM fructose and/or Pyrimidinopyrimidine, and were subjected to the GC-MS analysis to measure fructose-1-phosphate. Data are presented as means ± S.D. from n = 3 independent experiments. Significance was calculated by the an unpaired, two-sided Student's *t*-test, *P*-values are shown in the panel. g. MDA-MB-231 and MCF-7 cells, which had been transfected with 1μg of Myc-tagged plasmid or 60 nM si-RNA, were treated with 5 mM fructose and/or Pyrimidinopyrimidine, and were subjected to the DCF-DA assay. 20 μM H_2O_2 was used as positive control. Data are presented as means ± S.D. from n = 3 independent experiments. Significance was calculated by an unpaired, two-sided Student's *t*-test, *P*-values are represented in the panel. All experiments were independently repeated at least three times.

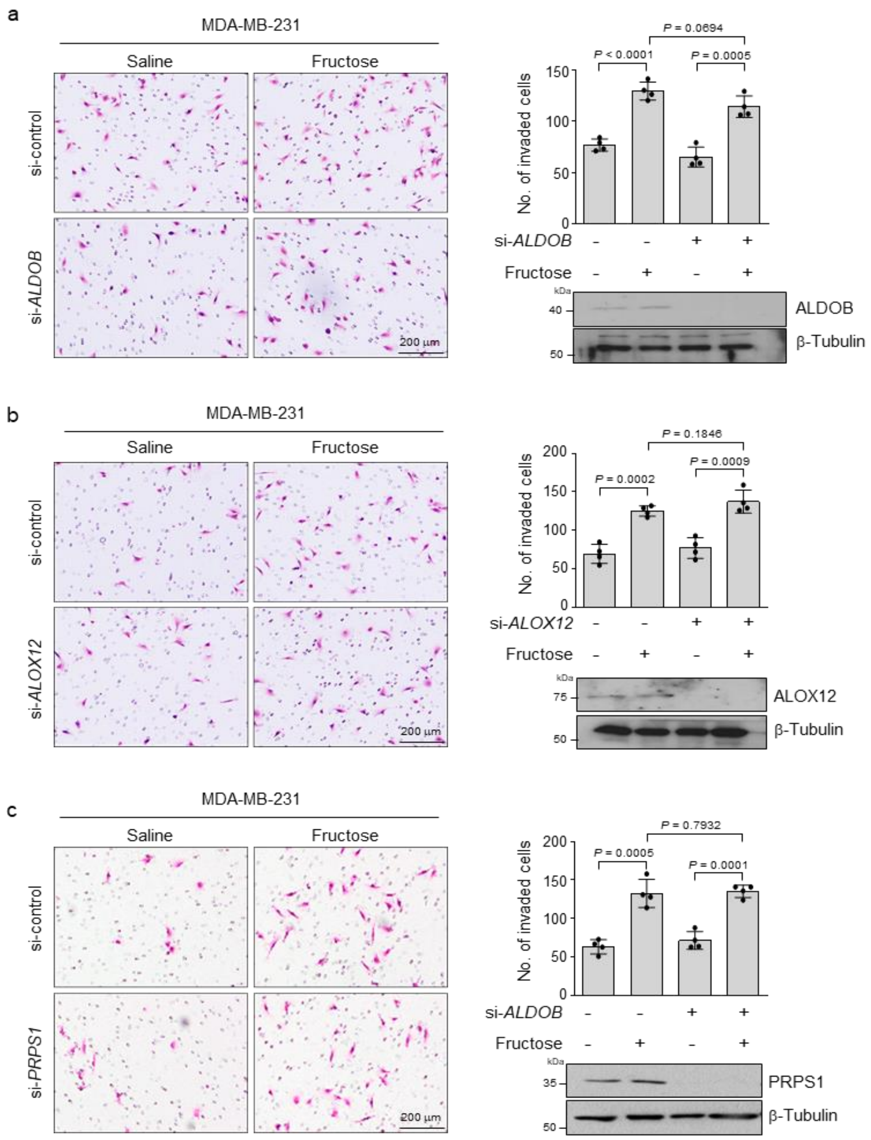

Supplementary Figure 4. Effect of ALDOB, ALOX12 or PRPS1 on invasion of fructose treated cancer cell. a. MDA-MB-231 cells, which had been transfected with 80 nM *ALDOB* si-RNA, were incubated with 5 mM fructose for 48 hours. Cells were subjected to Matrigel-coated invasion assay (means ± S.D. from n = 4 independent experiments). b. MDA-MB-231 cells, which had been transfected with 60 nM *ALOX12* si-RNA, were incubated with 5 mM fructose for 48 hours. Cells were subjected to Matrigel-coated invasion assay (means ± S.D. from n = 4 independent experiments). c. MDA-MB-231 cells, which had been transfected with 60 nM *PRPS1* si-RNA, were incubated with 5 mM fructose for 48 hours. Cells were subjected to Matrigel-coated invasion assay (means ± S.D. from n = 4 independent experiments). In a-c, statistical significance was calculated by an unpaired, two-sided Student's *t*-test.

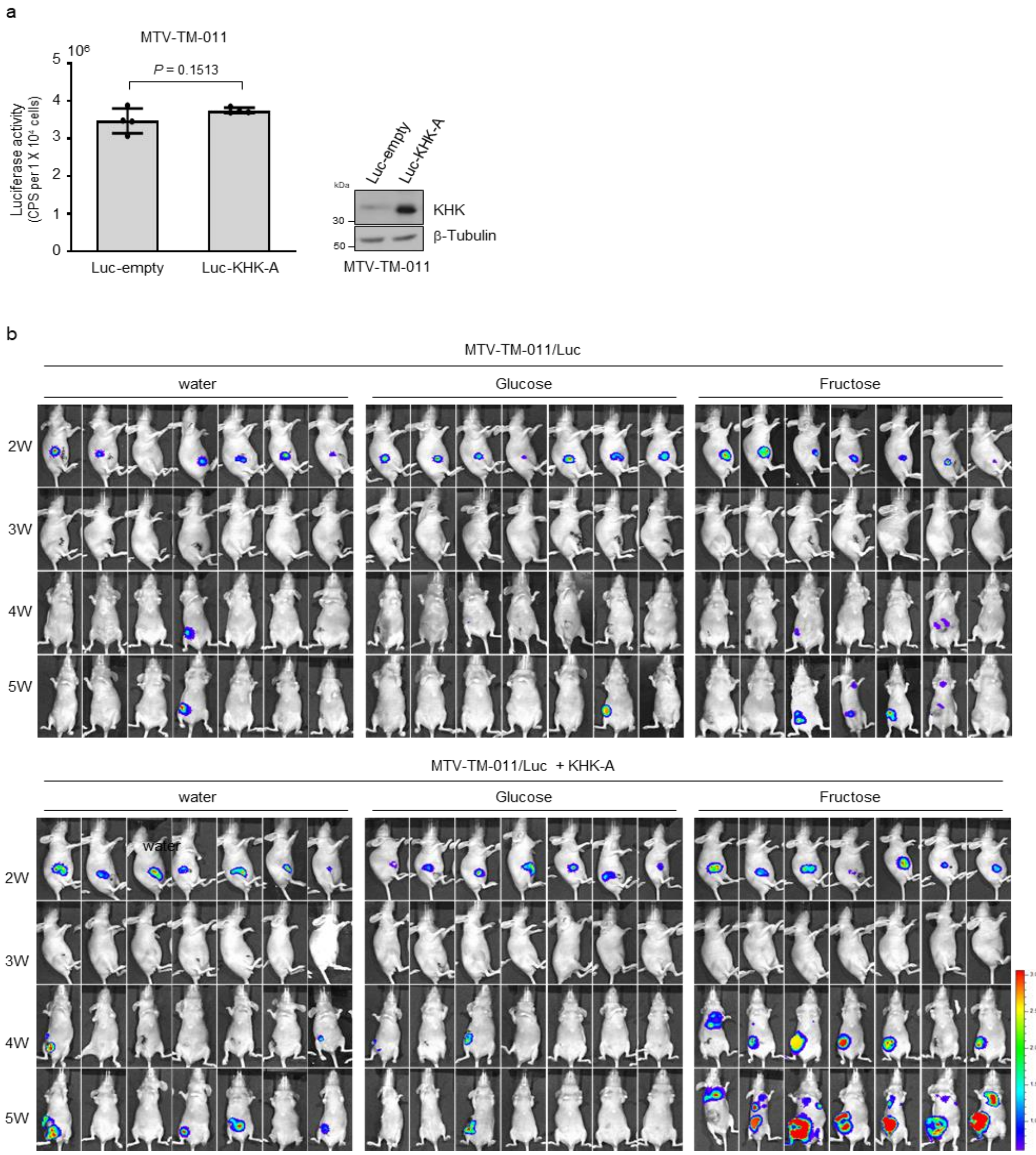

Supplementary Figure 5. KHK-A promotes the fructose-induced metastasis in *in vivo*. a. Establishment of stable cell lines. MTV-TM-011 cells were transfected with the Luciferase-IRES-GFP or Luciferase-IRES-KHK-A plasmid, and transfected cells were selected using G418. The expressions of luciferase and KHK-A were checked by luminometry and Western blotting respectively. (means ± S.D. from n = 4 independent experiments, and statistical significance was calculated by an unpaired, two-sided Student's t-test). b. Bioluminescence images of live mice were taken weekly using Xenogen IVIS 100, related to Figure 2d.

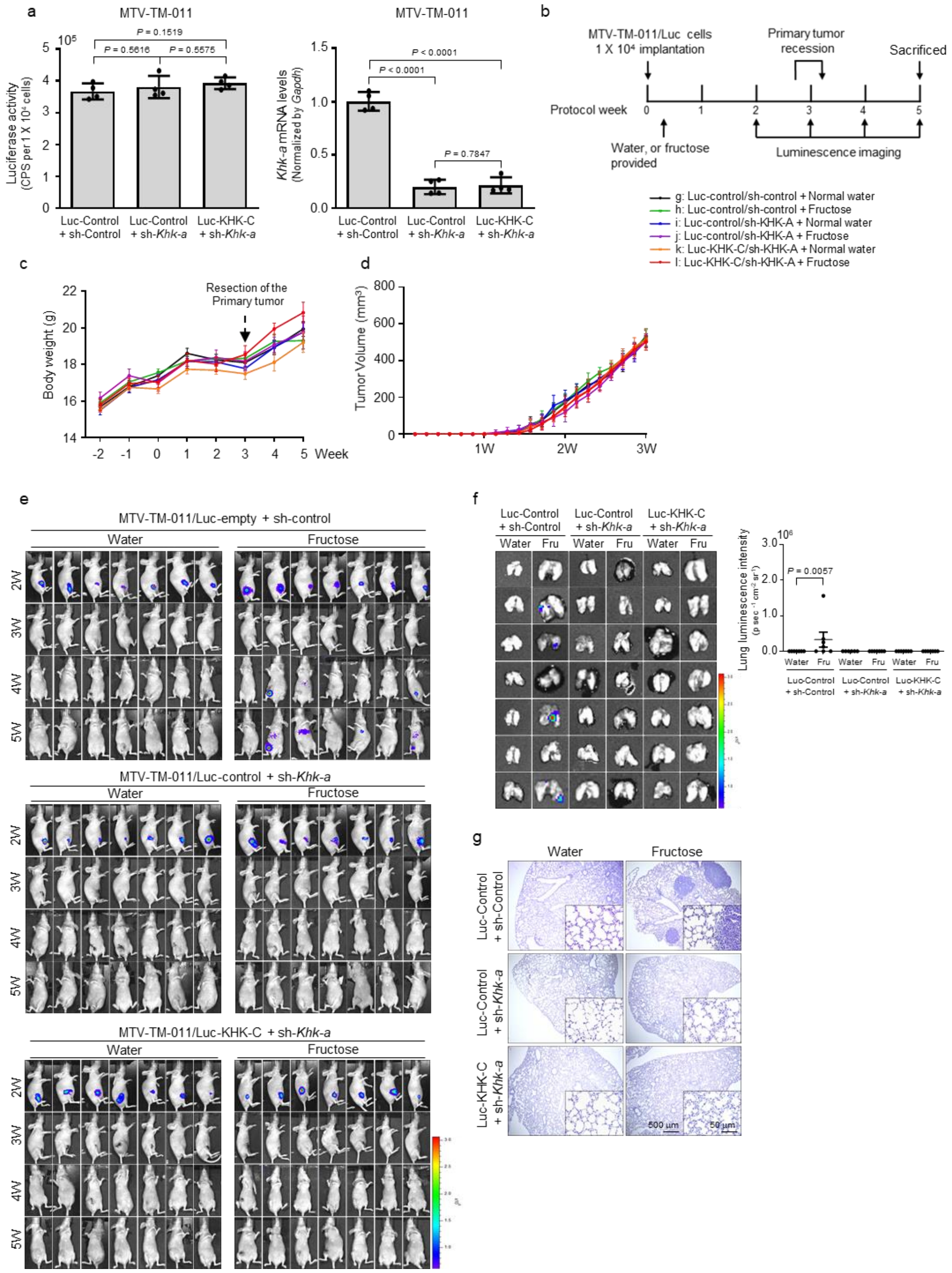

Supplementary Figure 6. KHK-C shows no effect on the fructose-induced metastasis. a. Establishment of stable cell lines. MTV-TM-011 cells were transfected with the Luciferase-IRES-GFP or Luciferase-IRES-KHK-C plasmid, and the cells were also infected with lentiviral shRNA targeting *Khk-a* or a scrambled sequence. Successfully transfected cells were selected using G418, and puromycin. The expression of luciferase and KHK-C were confirmed by luminometry, and silencing of endogenous

Khk-a were checked by qRT-PCR. Data are presented as means ± S.D. from $n = 4$ independent experiments. Significance was calculated by an unpaired, two-sided Student's *t*-test. b. Schematic diagram for the breast cancer xenograft study. MTV-TM-011 stable cells expressing luciferase and/or sh-*Khk-a* and/or KHK-C were implanted into mammary pads of mice. Tumor-bearing mice were fed with water, or 15% fructose. Primary tumors were removed when the tumor volumes reached 500 - 600 mm^3, and metastatic tumors were observed weekly by luminascence imaging. c. Body weights of mice were checked once a week and expressed as the means ± S.D. from $n = 7$ biologically independent mice per group. d**.** The volumes of breast tumors were monitored daily using calipers and expressed as the means ± S.D. The condition of each experimental group is described in the right panel. ($n = 7$ biologically independent mice per group) e. Bioluminescence images of tumor-bearing mice were taken weekly using Xenogen IVIS 100. The color bar represents bioluminescence intensity counts. f. On the 5th week after cancer graft, the organs were excised from mice. Bioluminescence images were captured in the lungs (left). Bioluminescence intensity (photones/sec/cm^2/sr) in the lungs was quantitatively analyzed (right). Data are presented as means ± S.D. from $n = 7$ lungs from biologically independent mice per group, and statistical significance was calculated by a two-sided Mann Whitney *U* test. g**.** Representative pictures of H&E-stained lungs.

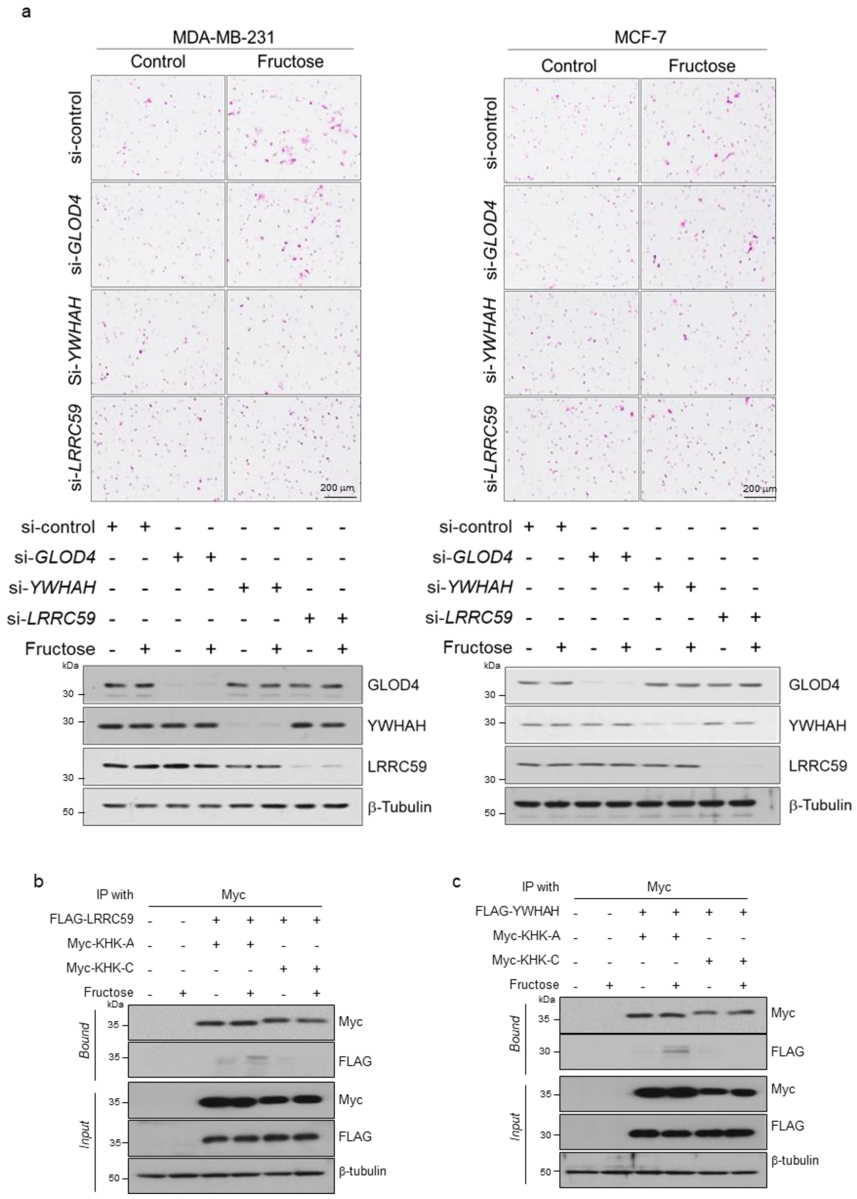

Supplementary Figure 7. Fructose promotes KHK-A binding to LRRC59 and YWHAH. a. Representative photographs of Matrigel-coated transwell invasion assays, related to Figure 3b. Western blots show the transfection efficiency in the corresponding cells. b. Interaction between ectopically expressed KHK-A/C and LRRC59. MDA-MB-231 cells were transfected with Myc-KHK-A/C and FLAG-LRRC59, and incubated with 5 mM fructose for 8 hours. The cell lysates were immunoprecipitated with anti-Myc, and immunoblotted with anti-FLAG. c. Interaction between ectopically expressed KHK-A/C and YWHAH. Transfected MDA-MB-231 cells were incubated with 5 mM fructose for 8 hours, and subjected to immunoprecipitation and immunoblotting. All experiments were independently repeated at least three times.

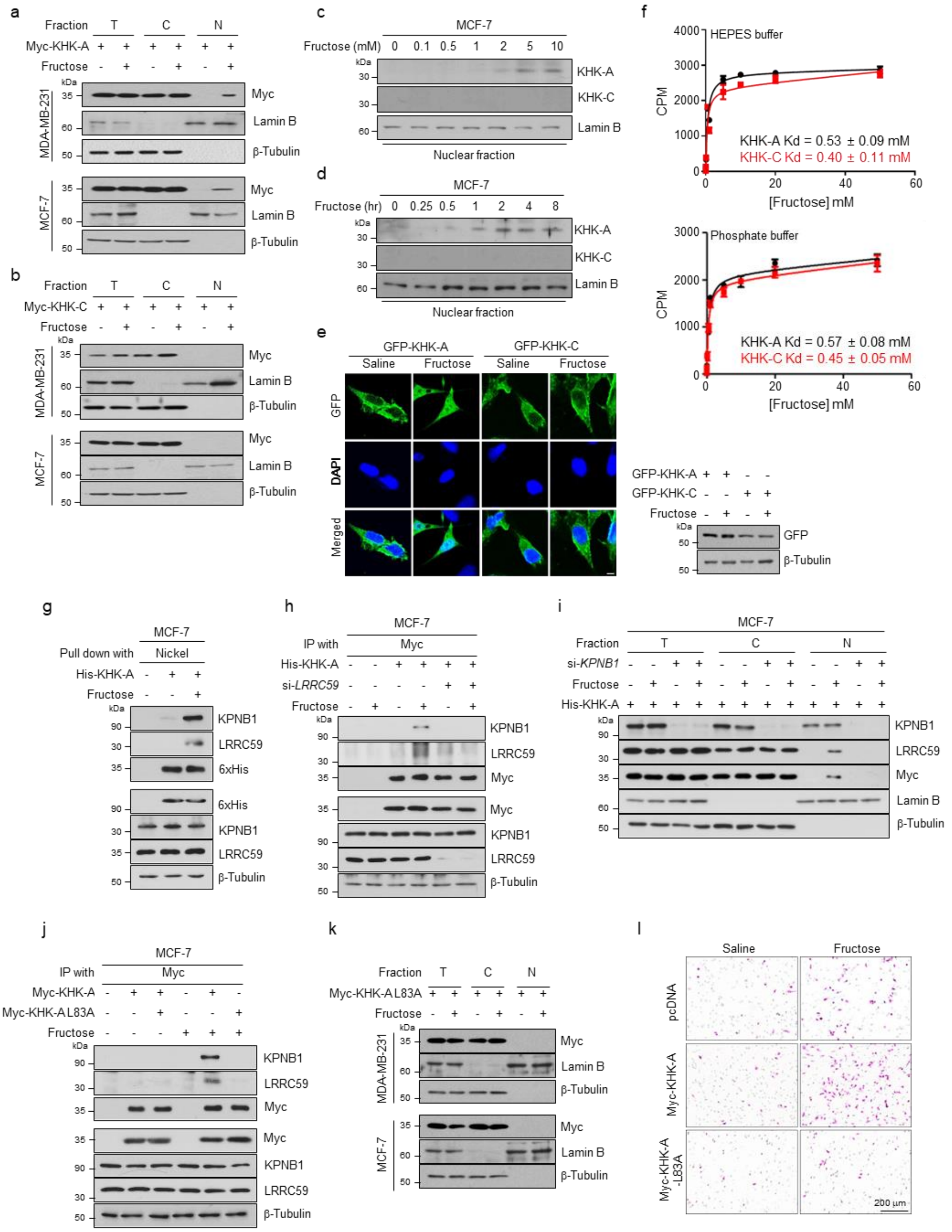

Supplementary Figure 8. Translocalization of KHK-A to the nucleus under fructose stimuli. a. Subcellular localization of KHK-A. MDA-MB-231 and MCF7 cells were transfected with Myc-KHK-A, and incubated with fructose. Total lysates (T) were fractionated to cytosolic (C) and nuclear (N) components. b. MDA-MB-231 and MCF-7 cells were transfected with Myc-KHK-C. The cell fractions were immunoblotted with indicated antibodies. c. MCF-7 cells were treated with fructose at the indicated concentrations for 2 hr. Nuclear KHK-A and KHK-C was subjected to immunoblotting. d.

MCF-7 cells were treated with 5 mM fructose for the indicated times. Nuclear KHK-A and KHK-C was immunoblotted. e. Immunofluorescence imaging of MDA-MB-231 cells expressing GFP-tagged KHK-A/C (green), and nuclei were counterstained with DAPI (blue). Scale bar represents 10 μm. f. Measurement of the dissociation constant (Kd) for fructose. Recombinant His-KHK-A and His-KHK-C were incubated with various concentrations of fructose in HEPES or phosphate buffer. The K_d values of KHK-A and KHK-C for fructose were calculated based on the binding-saturation model. Data are presented as means ± S.D. from $n = 3$ independent experiments. g. MCF-7 cells expressing His-KHK-A, were treated with fructose. His-KHK-A was pulled down using Nickel-NTA, and the co-precipitated LRRC59 and KPNB1 were identified by immunoblotting. h. MCF-7 cells, which had been co-transfected with Myc-KHK-A and si-*LRRC59*. Myc-KHK-A was immunoprecipitated and co-precipitaed proteins were immunoblotted. i. MCF-7 cells, which had been co-transfected with His-KHK-A and si-*KPNB1*, were fractioned. The cell fractions were immunoblotted to check the subcellular location of KHK-A and LRRC59. j. MCF-7 cells were transfected with Myc-KHK-A or Myc-KHK-A L83A. The cell lysates were immunoprecipitated with anti-Myc and immunoblotted with anti-LRRC59 or anti-KPNB1. k. Subcellular localization of KHK-A L83A. MDA-MB-231 and MCF7 cells were transfected with Myc-KHK-A L83A. l. MDA-MB-231 cells, which had been transfected with Myc-KHK-A wild type or -L83A plasmid, were treated with 5 mM fructose for 48 hours, and subjected to invasion assay. All experiments were independently repeated at least three times. In a, b, d, g, h, i, j, k, transfected breast cancer cells were incubated with 5 mM fructose for 8 hours.

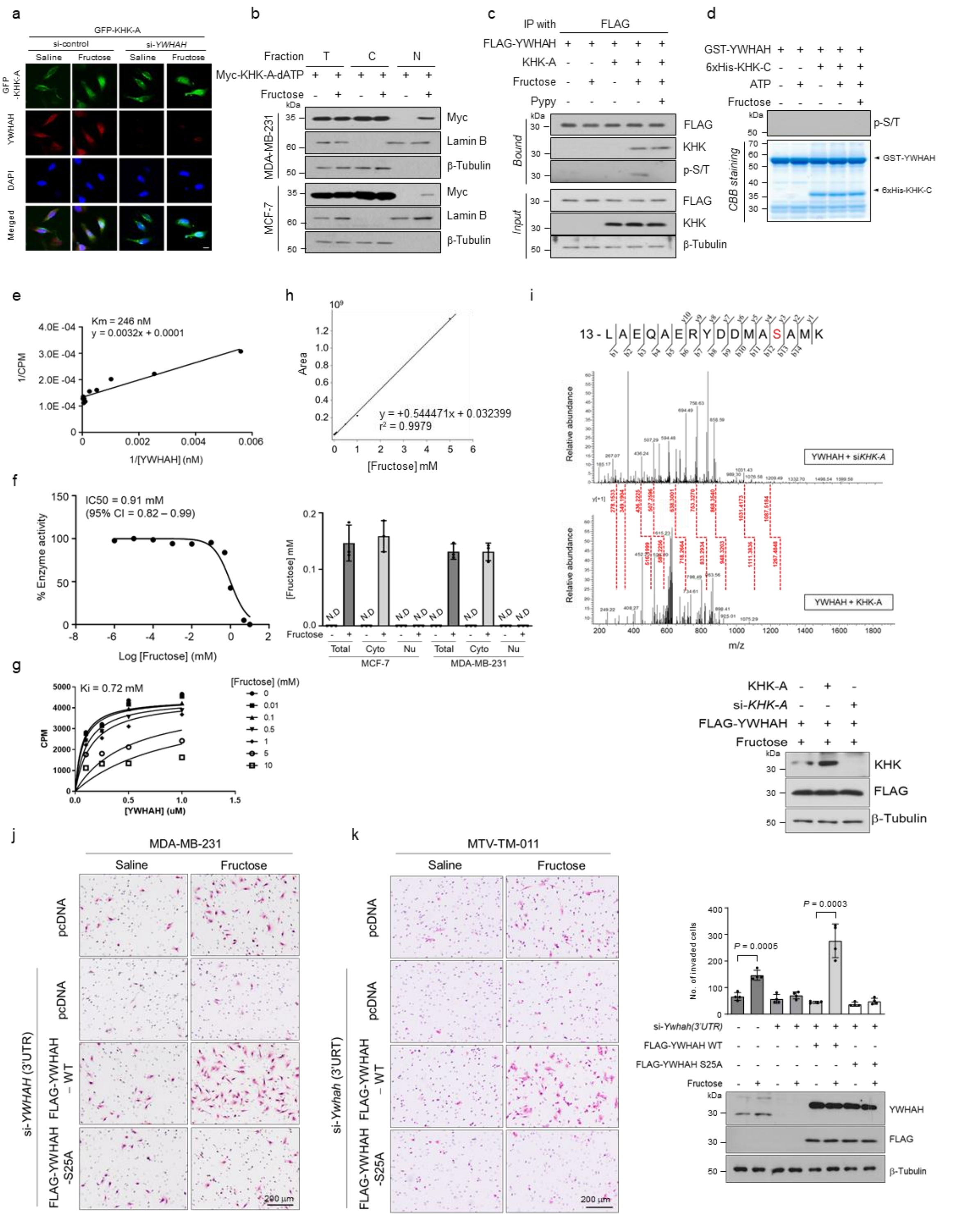

Supplementary Figure 9. KHK-A functions as a nuclear protein kinase. a. Immunofluorescence imaging of KHK-A (green) and YWHAH (red). MDA-MB-231 cells were incubated with 5 mM fructose for 8 hours. Scale bar represent 10 µm. b. MDA-MB-231 and MCF7 cells were transfected with Myc-KHK-A_dATP, and incubated with 5 mM fructose for 8 hours, and total lysates (T) were fractionated to

cytosolic (C) and nuclear (N) components. c. MDA-MB-231 stable cells expressing KHK-A were transfected with FLAG-YWHAH, were incubated with 5 mM fructose and/or 400 nM Pypy for 8 hours. d. *in vitro* kinase assay. Recombinant GST-YWHAH and His-KHK-C were co-incubated without or with 1 mM fructose in a kinase buffer. The Ser/Thr-phosphorylation of YWHAH was confirmed by immunoblotting. e. The Km value of GST-KHK-A for GST-YWHAH with representative plotting of 1/CPM versus 1/[YWHAH] was analyzed. f. The IC50 of KHK-A for YWHAH was calculated by plotting of percentage of KHK-A enzyme activity versus log[fructose]. g. The K_i of fructose for the KHK-A-mediated YWHAH phosphorylation was fitted with the competitive inhibition model. h. MCF-7 and MDA-MB-231 cells were treated with 5 mM fructose for 4 hours. Fructose concentration in each total, cytoplasmic, and nuclear fraction were calculated based on a standard curve which is generated by known fructose concentration. Measured fructose levels were normalized by packed cell volume. Data are presented as means ± S.D. from $n = 3$ independent experiments. i. Mass spectrometric analysis for phosphorylation site of YWHAH in MCF-7 cell lysate. The cell lysates were subjected to LC-MS/MS analysis. j. Effect of YWHAH or S25A mutant proteins on cell invasion potential. After incubated with 5 mM fructose for 48 hours, cells were subjected to Matrigel invasion assay, related to Figure 5g. k. Endogenous YWHAH was removed from MTV-TM-011 cells using an siRNA targeting the 3′-UTR region of YWHAH mRNA, and FLAG-YWHAH or S25A mutant proteins were restored in the cells. After incubated with 5 mM fructose for 48 hours, cells were subjected to Matrigel invasion assay (means ± S.D. from $n = 4$ independent experiments). Significance was calculated by an unpaired, two-sided Student's *t*-test.

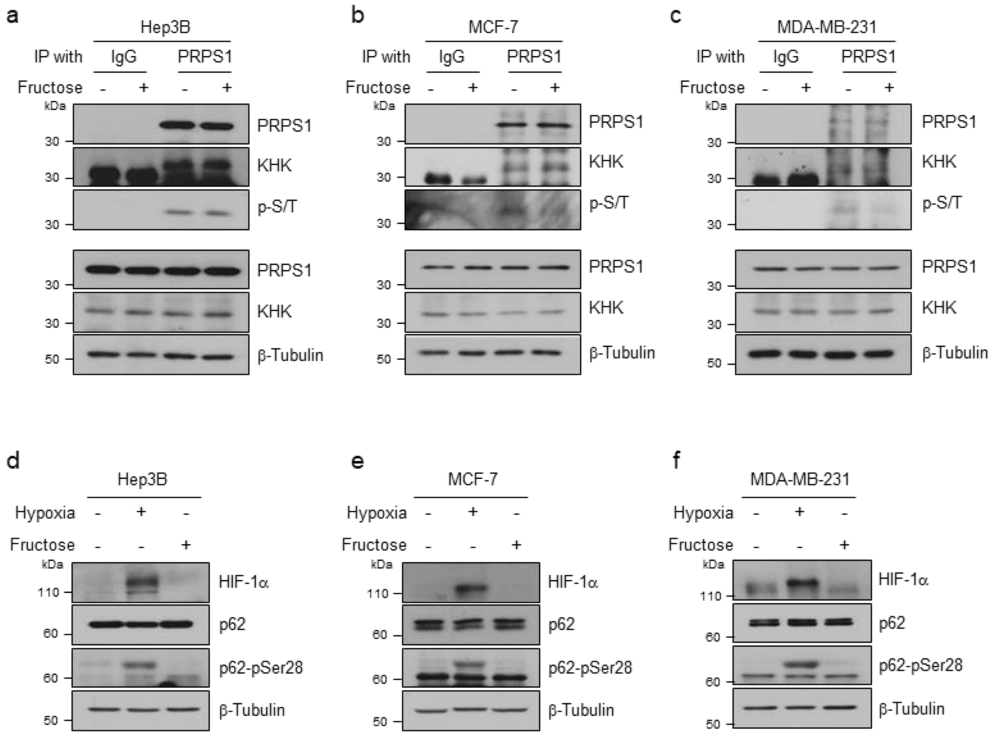

Supplementary Figure 10. Validation of kinase function of KHK-A. a. Hep3B, b. MCF-7 and c. MDA-MB-231 cells were incubated with 5 mM fructose, and cell lysates were subjected to immunoprecipitation with PRPS1 antibody (or IgG), and immunoblotted with anti-KHK or anti-phospho-S/T antibody. d. Hep3B, e. MCF-7 and f. MDA-MB-231 cells were incubated under hypoxia (1% O_2) or 5 mM fructose for 6 hours. Total lysates were subjected to Western blotting with the indicated antibodies. In a-f, data represent one out of three experiments.

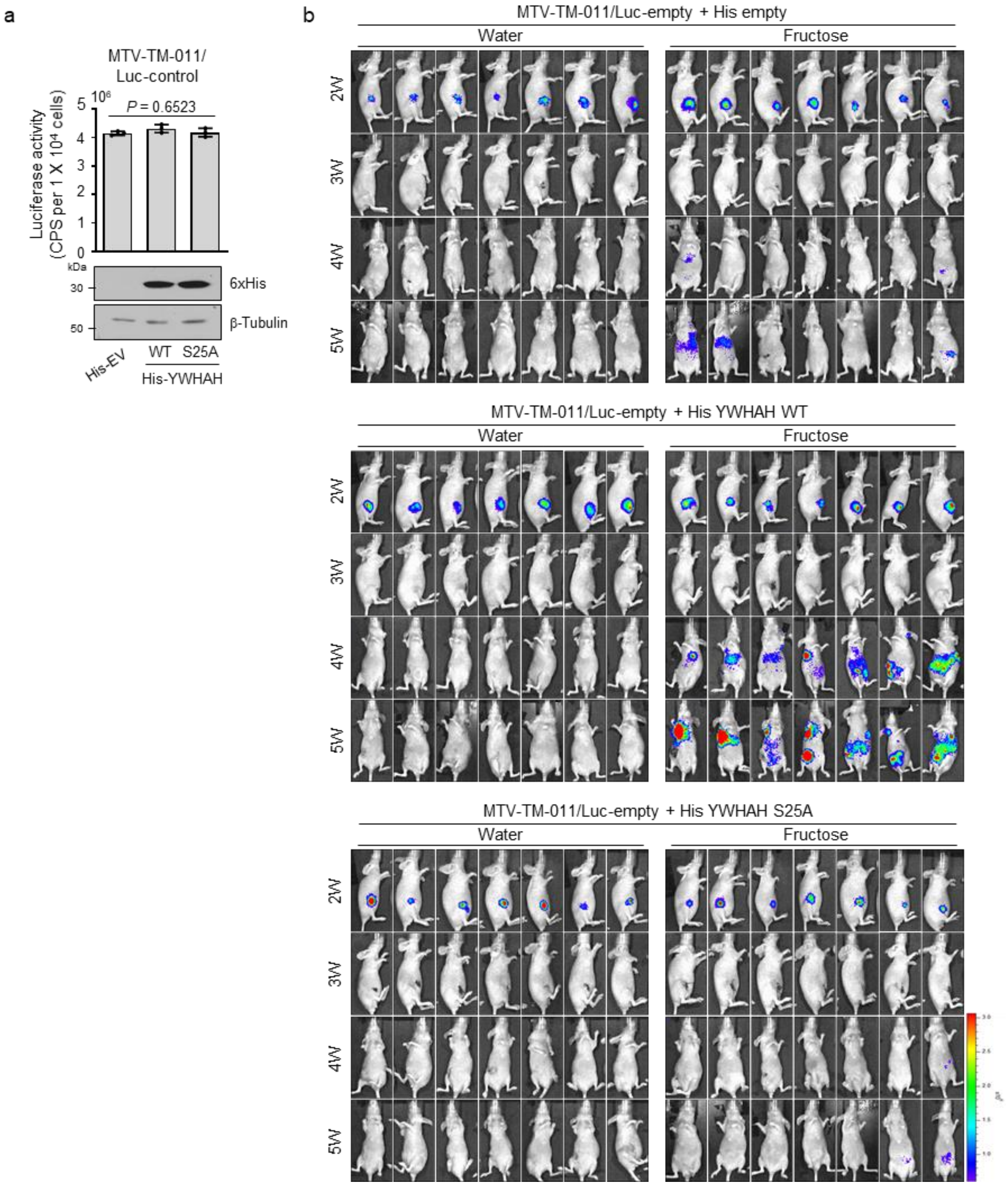

Supplementary Figure 11. YWHAH-pSer25 promotes breast cancer metastasis in fructose-fed mice. a. Establishment of stable cell lines. MTV-TM-011 cells were transfected with the Luciferase-IRES-GFP, and His-empty vector, His-YWHAH-WT or His-YWHAH-S25A plasmid, and transfected cells were double selected using G418 and zeocin. The expressions of luciferase and His-YWHAH-WT and His-YWHAH-S25A were checked by luminometry and Western blotting respectively. (means ± S.D. from *n* = 3 independent experiments, statistical significance was calculated by one way ANOVA test). b. Bioluminescence images of live mice were taken weekly using Xenogen IVIS 100, related to Figure 6d.

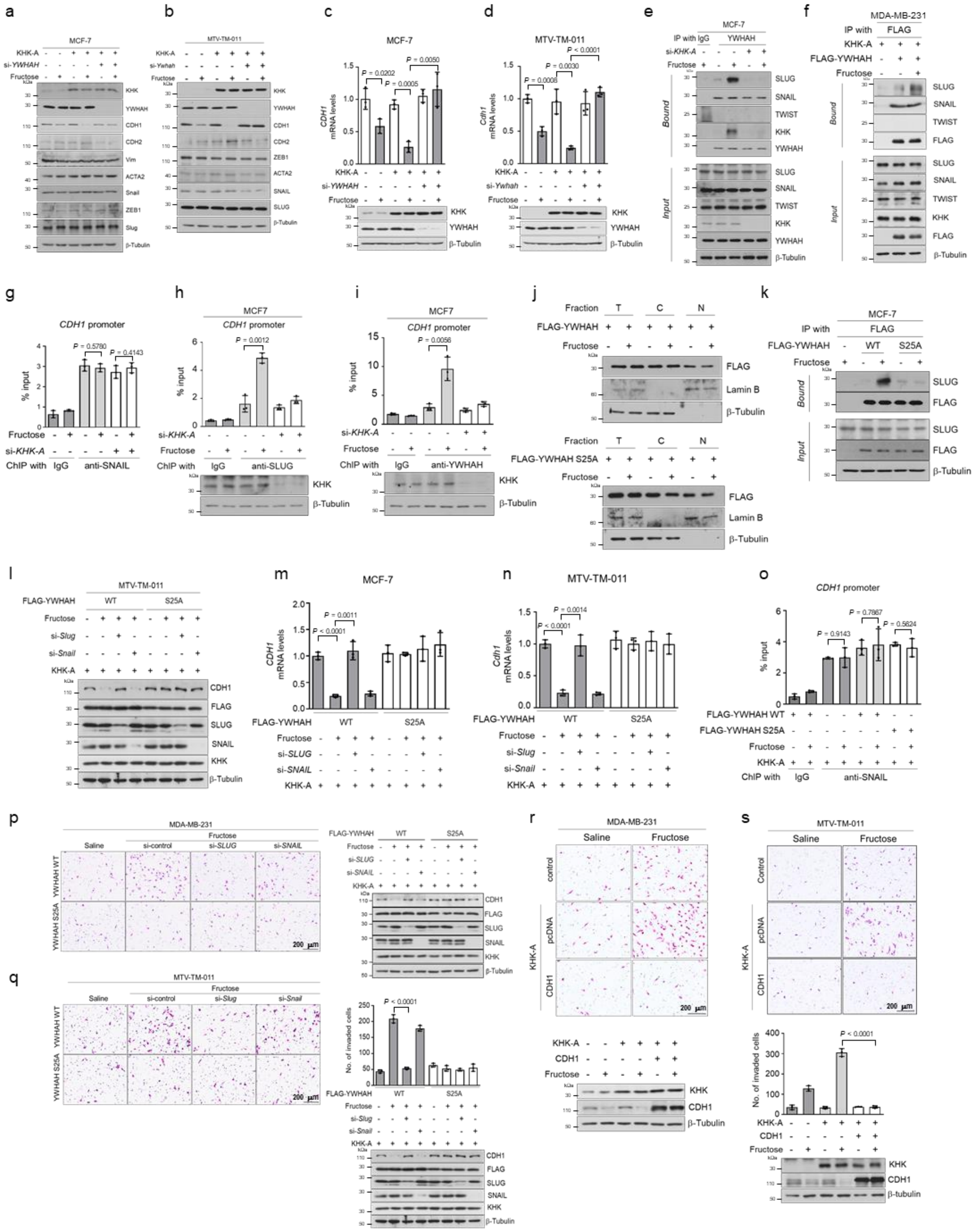

Suppementary Figure 12. YWHAH -pSer25 represses transcriptional activity of CDH1. a. The EMT marker analysis. MCF-7 cells stably expressing KHK-A were transfected with si-*YWHAH*. b. The EMT marker analysis. MTV-TM-011 cells stably expressing KHK-A were transfected with si-*Ywhah*. c. The mRNA levels of *CDH1* were analyzed in MCF-7 cells. d. The mRNA levels of *Cdh1* were analyzed in

MTV-TM-011 cells. e. Transfected MCF-7 cells were subjected to immunoprecipitation and immunoblotting. f. MDA-MB-231 cells stably expressing KHK-A were transfected with FLAG-YWHAH. g. MDA-MB-231 cells were transfected with si-*KHK-A* were subjected to ChIP-qPCR. h. MCF-7 cells were transfected with si-*KHK-A*, and cells were subjected to ChIP-qPCR. i. MCF-7 cells were transfected with si-*KHK-A*, and cells were subjected to ChIP-qPCR. j. MDA-MB-231 cells were transfected with FLAG-YWHAH wild type or S25A mutant plasmid, and incubated with 5 mM fructose for 8 hours. k. MCF-7 cells, which had been transfected with Flag-YWHAH (or S25A), were subjected to immunoprecipitation and immunoblotting. l. MTV-TM-011 cells, which had been transfected as indicated, were subjected to immunoblotting. m. The mRNA levels of *CDH1* were analyzed by RT-qPCR in MCF-7 cells. n. The mRNA levels of *Cdh1* were analyzed in MTV-TM-011 cells. o. Transfected MDA-MB-231 cells were subjected to immunoprecipitated with anti-SNAIL antibody and the SNAIL-bound *CDH1* promoter was quantified by RT-qPCR. p. Transfected MDA-MB-231 cells were subjected to Matrigel invasion assay. q. Transfected MTV-TM-011 cells were subjected to Matrigel invasion assay. r. MDA-MB-231 cells were co-transfected with KHK-A and CDH1, were subjected to Matrigel invasion assay, related to Figure 6l. s. Transfected MTV-TM-011 cells were subjected to Matrigel invasion assay and immunoblotting. All experiments were independently repeated at least three times. In a, b, c, d, l, m, n, p, q, r, s, transfected cells were incubated with 5 mM fructose for 48 hours, and in e, f, g, h, i, k, o, transfected cells were incubated with 5 mM fructose for 24 hours. In c,d,g,h,i,m,n,o,q,s, data are presented as means ± S.D. from $n = 3$ independent experiments. Statistical significance was calculated by an unpaired, two-sided, Student's *t*-test. *P*-values are represented in each panel.

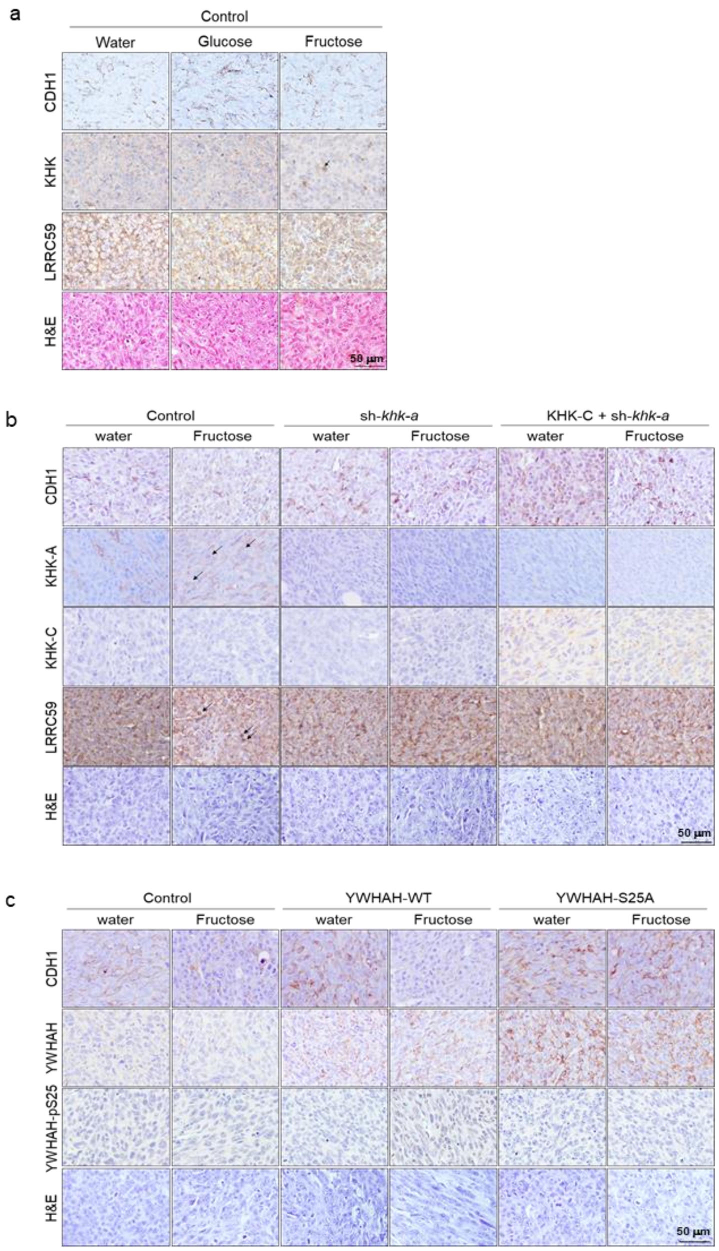

Supplementary Figure 13. a. The tumor sections from the in vivo experiment which were presented in Fig 2, were immunostained with CDH1, KHK, and LRRC59 antibodies. Also the slides were stained with Hematoxylin and Eosin. b. The primary breast tumor section from the mice presented in supplementary fig 6, were immunostained with CDH1, KHK-A, KHK-C, and LRRC59 antibodies. The slides were also stained with Hematoxylin and Eosin. c. The primary breast cancer masses sections from the mice presented in Fig 6, were immunostained with YWHAH, YWHAH-pS25, and CDH1. Also the slides were stained with Hematocylin and Eosin. In a-c, data represent one out of three independent experiments.

1f

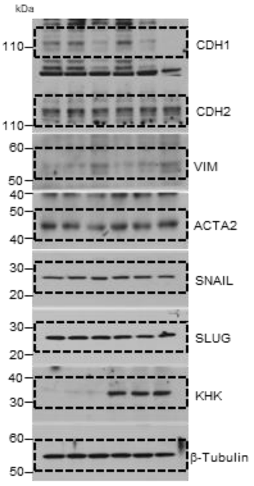

3c

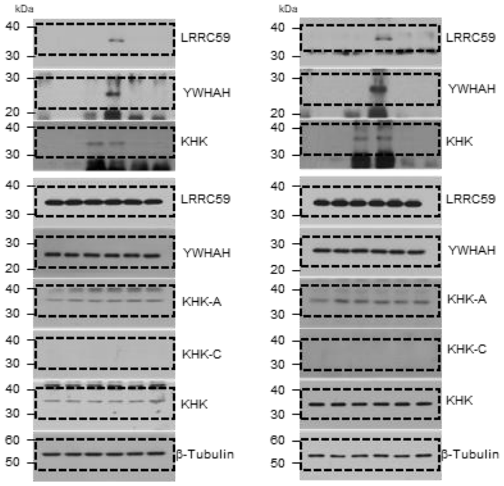

3d

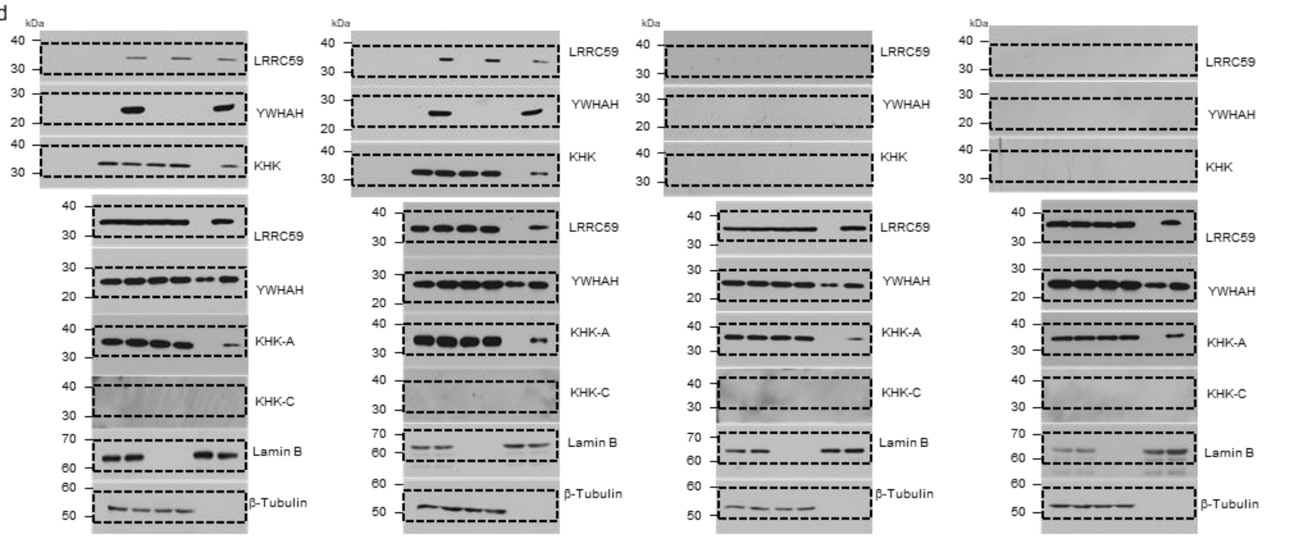

4a

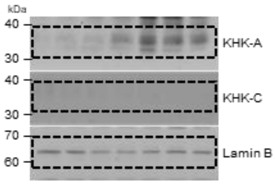

4b

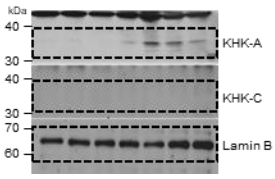

4d

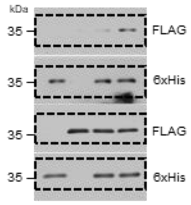

4e

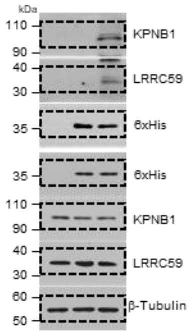

4f

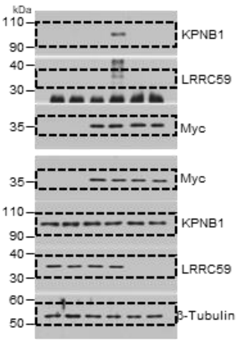

4g

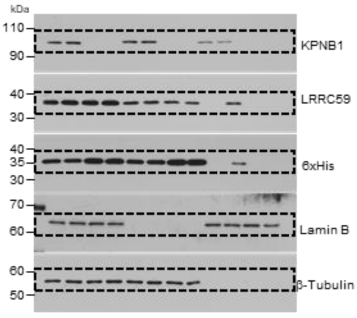

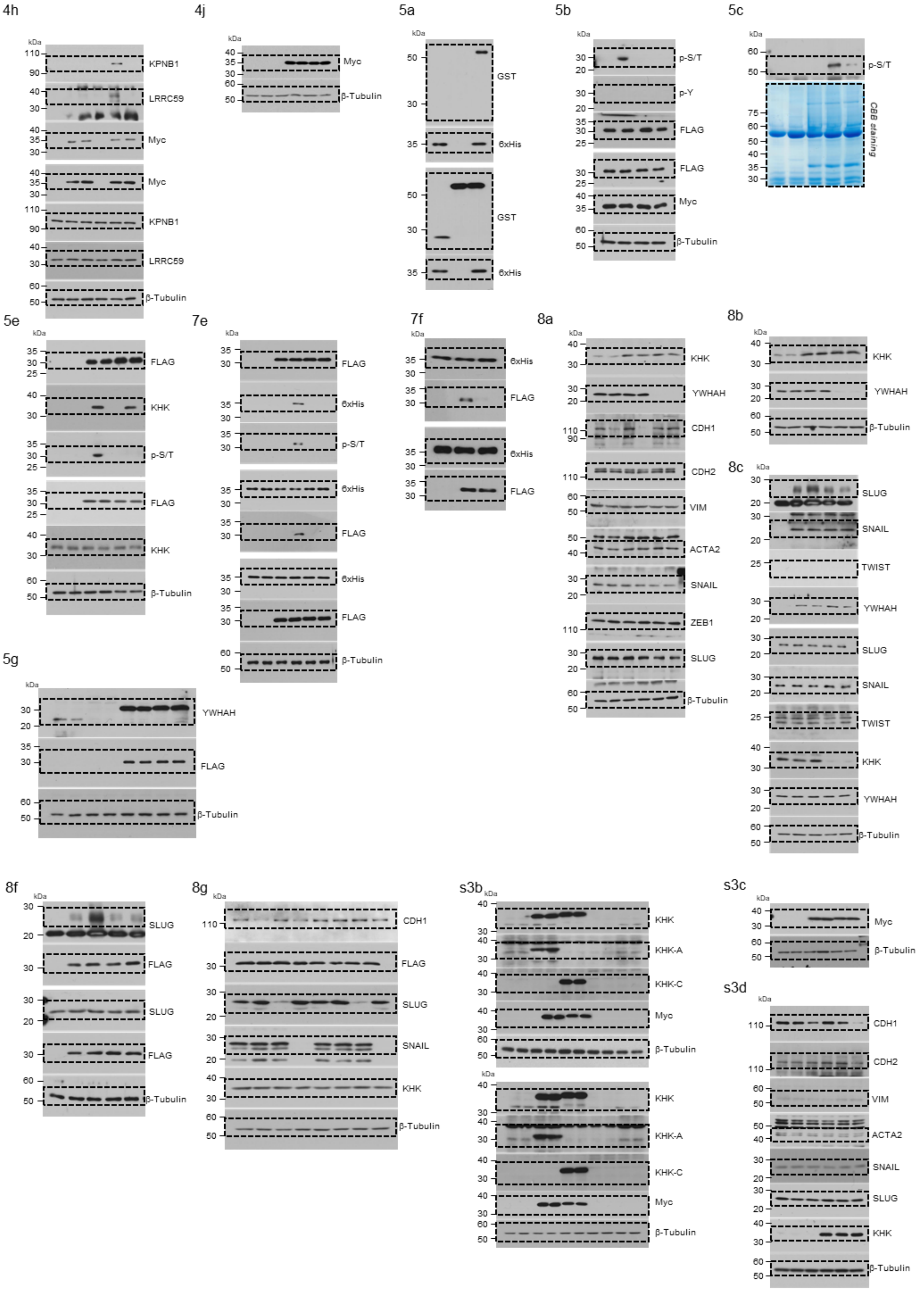
4h
kDa
110 90 KPNB1
40 30 LRRC59
40 35 30 Myc
40 35 30 Myc
110 90 KPNB1
40 30 LRRC59
60 50 β-Tubulin
4j
kDa
40 35 30 Myc
60 50 β-Tubulin
5a
kDa
50 30 GST
35 6xHis
50 30 GST
35 6xHis
5b
kDa
30 20 p-S/T
30 20 p-Y
35 30 25 FLAG
30 25 FLAG
40 35 Myc
60 50 β-Tubulin
5c
kDa
60 50 p-S/T
75 60 50 40 35 30 CBB staining
5e
kDa
35 30 25 FLAG
40 30 KHK
35 30 25 p-S/T
35 30 25 FLAG
40 30 KHK
60 50 β-Tubulin
7e
kDa
35 30 FLAG
35 30 6xHis
35 30 p-S/T
35 30 6xHis
35 30 FLAG
35 30 6xHis
35 30 FLAG
60 50 β-Tubulin
7f
kDa
35 30 6xHis
35 30 FLAG
35 30 6xHis
35 30 FLAG
8a
kDa
40 30 KHK
30 20 YWHAH
110 90 CDH1
110 CDH2
60 50 VIM
50 40 ACTA2
30 20 SNAIL
110 ZEB1
30 20 SLUG
60 50 β-Tubulin
8b
kDa
40 30 KHK
30 20 YWHAH
60 50 β-Tubulin
8c
kDa
30 20 SLUG
30 20 SNAIL
25 TWIST
30 20 YWHAH
30 20 SLUG
30 20 SNAIL
25 TWIST
40 30 KHK
30 20 YWHAH
60 50 β-Tubulin
5g
kDa
30 20 YWHAH
35 30 FLAG
60 50 β-Tubulin
8f
kDa
30 20 SLUG
30 FLAG
30 20 SLUG
30 FLAG
60 50 β-Tubulin
8g
kDa
110 CDH1
30 FLAG
30 20 SLUG
30 20 SNAIL
40 30 KHK
60 50 β-Tubulin
s3b
kDa
40 30 KHK
40 30 KHK-A
40 30 KHK-C
40 30 Myc
60 50 β-Tubulin
kDa
40 30 KHK
40 30 KHK-A
40 30 KHK-C
40 30 Myc
60 50 β-Tubulin
s3c
kDa
40 30 Myc
60 50 β-Tubulin
s3d
kDa
110 CDH1
110 CDH2
60 50 VIM
50 40 ACTA2
30 20 SNAIL
30 20 SLUG
40 30 KHK
60 50 β-Tubulin

s3e

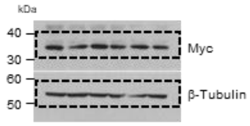

s3f

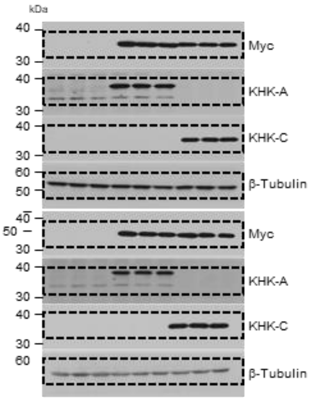

s3g

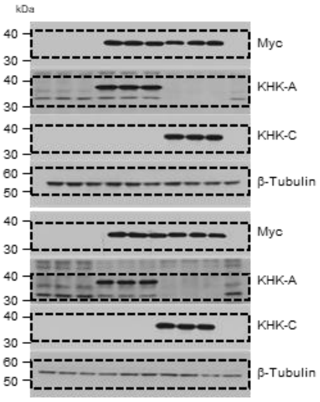

s4a

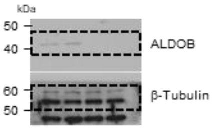

s4b

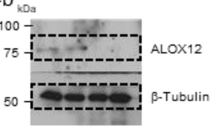

s4c

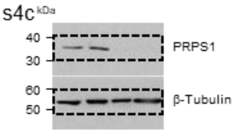

s5a

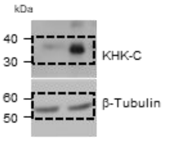

s7a

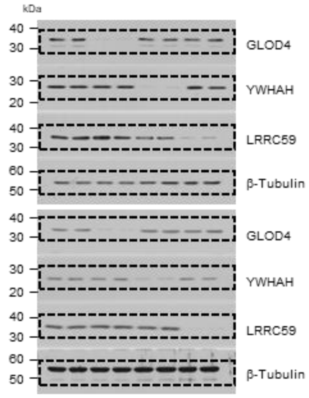

s7b

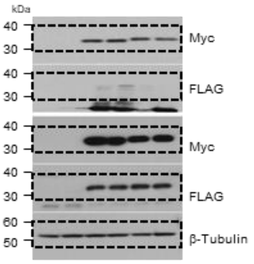

s7c

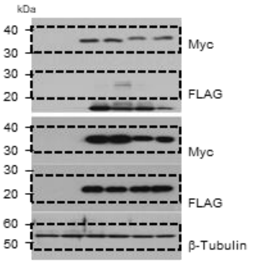

s8a

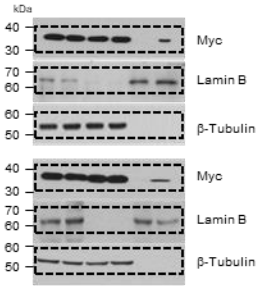

s8b

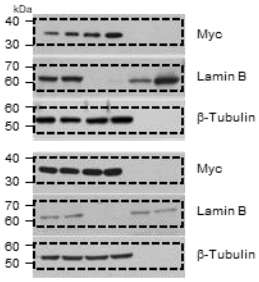

s8c

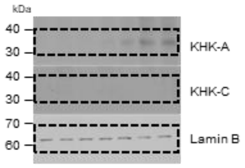

s8d

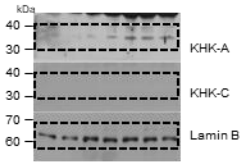

s8e

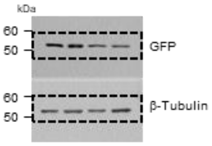

s8g

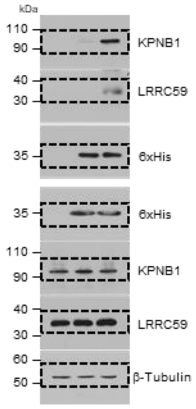

s8h

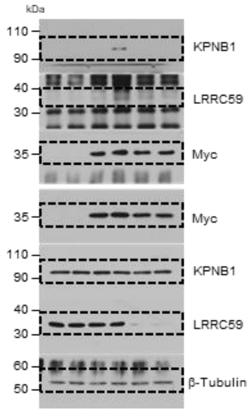

s8i

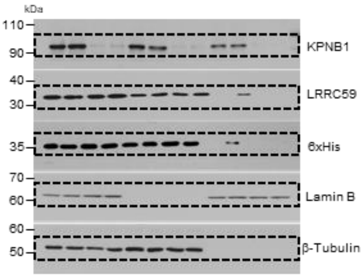

s8j

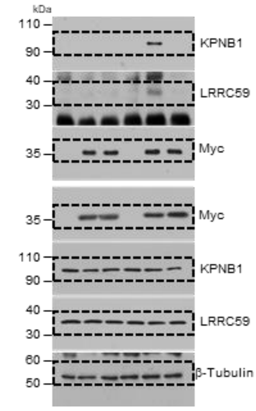

s8k

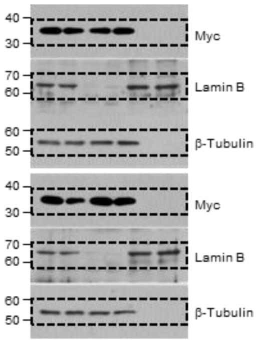

s9b

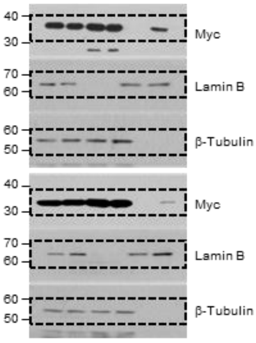

s9c

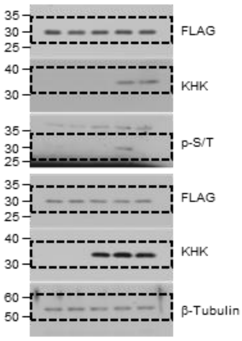

s9d

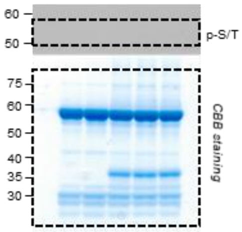

s9i

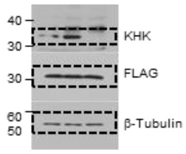

s9k

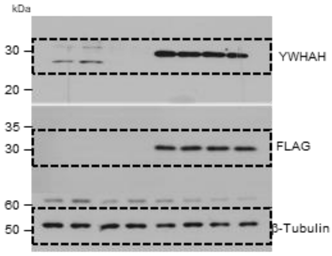

s10a

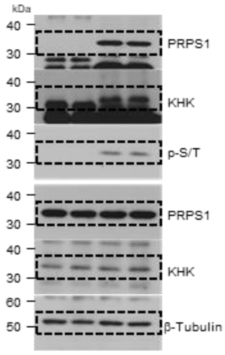

s10b

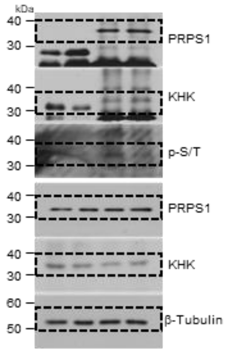

s10c

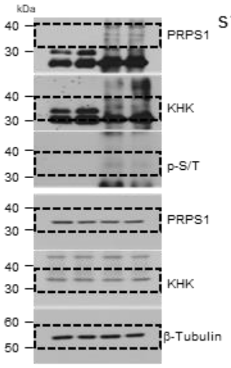

s10d

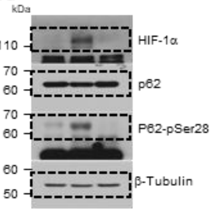

s12a

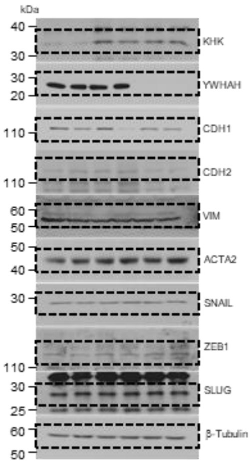

s12b

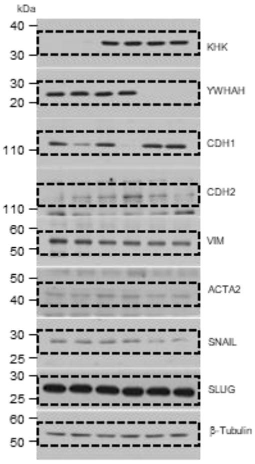

s10e

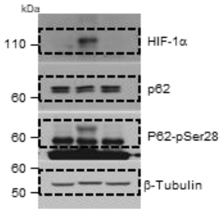

s10f

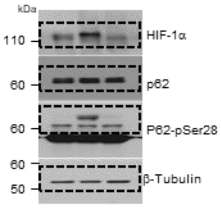

s11a

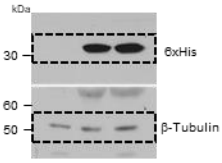

s12c

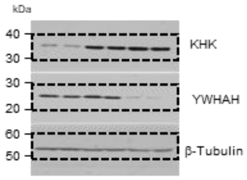

s12e

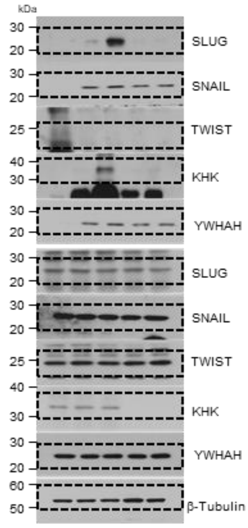

s12f

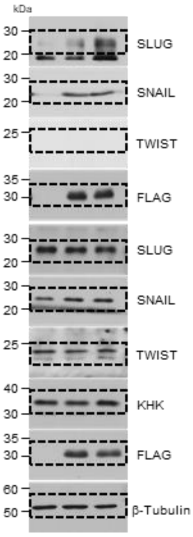

s12h

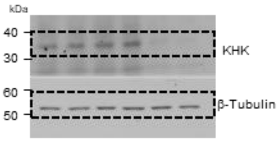

s12j

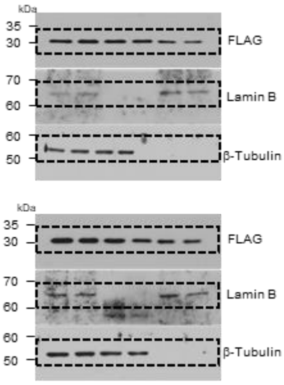

s12d

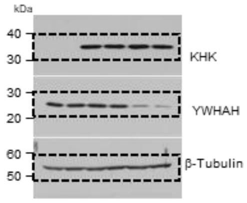

s12i

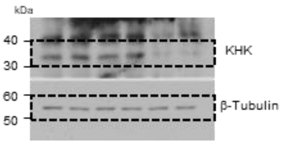

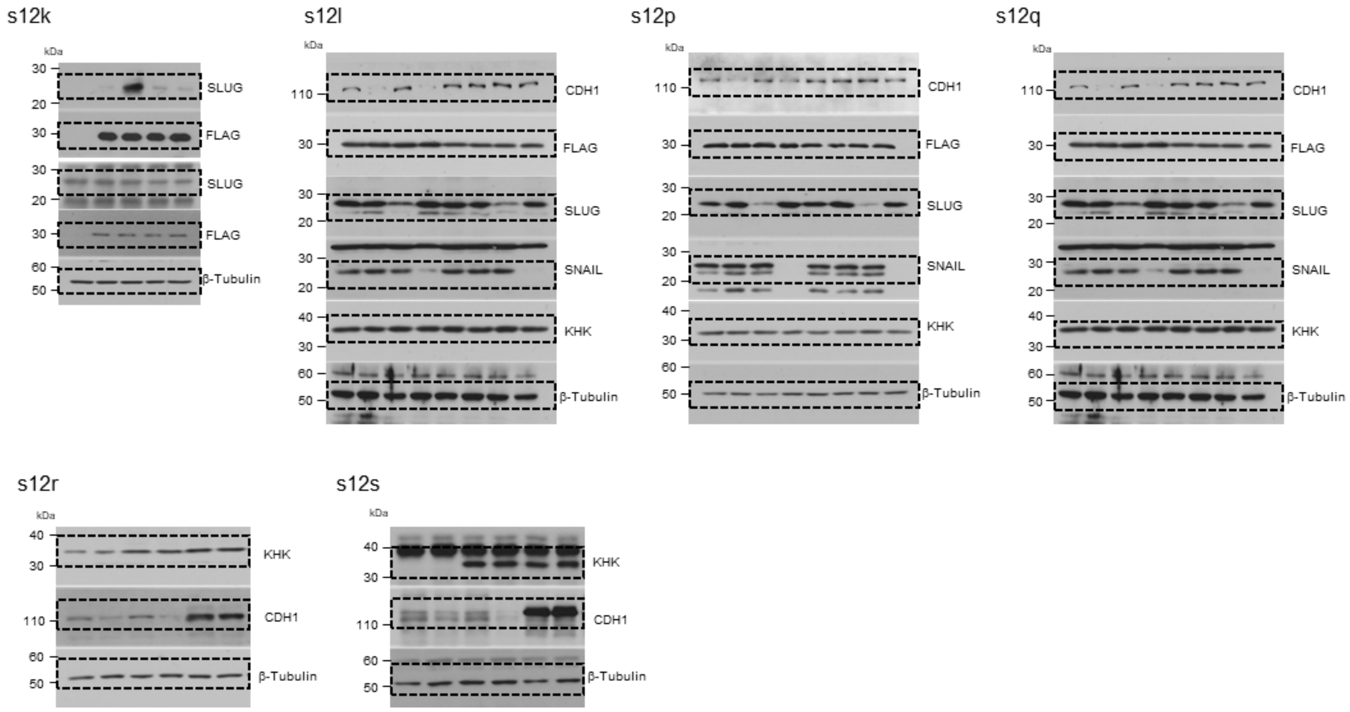

Supplementary Figure 14. Original images of western blots in the study. Uncropped blots represented with protein name and molecular weight markers. All blots are listed in the following order 1f; 3c, d; 4a, b, d, e, f, g, h, j, 5a, b, c, e, g; 7e, f; 8a, b, c, f, g; and Supplementary figures 3b, c, d, e, f, g; 4a, b, c; 5a; 7a, b, c; 8a, b, c, d, e, g, h, i, j, k; 9b, c, d, i, k; 10a, b, c, d, e, f; 11a; 12a, b, c, d, e, f, h, i, j, k, l, p, q, r, s.

a

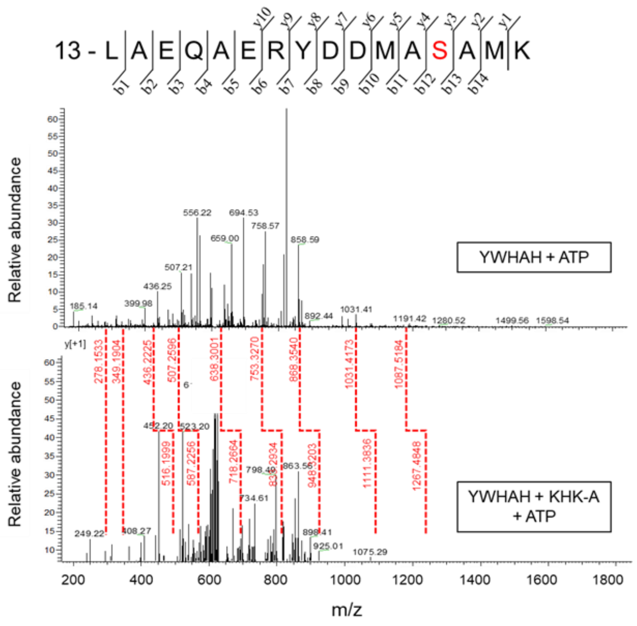

b

#1	b⁺	b^{2+}	b^{3+}	Seq.	y⁺	y^{2+}	y^{3+}	#2
1	114.09135	57.54931	38.70197	L				16
2	185.12847	93.06787	62.38101	A	1715.73644	858.37186	572.58366	15
3	314.17107	157.58917	105.39521	E	1644.69932	822.85330	548.90462	14
4	442.22965	221.61846	148.08140	Q	1515.65672	758.33200	505.89042	13
5	513.26677	257.13702	171.76044	A	1387.59814	694.30271	463.20423	12
6	642.30937	321.65832	214.77464	E	1316.56102	658.78415	439.52519	11
7	798.41049	399.70888	266.80835	R	1187.51842	594.26285	396.51099	10
8	961.47381	481.24054	321.16279	Y	1031.41730	516.21229	344.47728	9
9	1076.50076	538.75402	359.50510	D	868.35398	434.68063	290.12284	8
10	1191.52771	596.26749	397.84742	D	753.32703	377.16715	251.78053	7
11	1322.56821	661.78774	441.52759	M	638.30008	319.65368	213.43821	6
12	1393.60533	697.30630	465.20663	A	507.25958	254.13343	169.75804	5
13	1480.63736	740.82232	494.21730	S	436.22246	218.61487	146.07900	4
14	1551.67448	776.34088	517.89634	A	349.19043	175.09885	117.06833	3
15	1682.71498	841.86113	561.57651	M	278.15331	139.58029	93.38929	2
16				K	147.11281	74.06004	49.70912	1

c

#1	b⁺	b^{2+}	b^{3+}	Seq.	y⁺	y^{2+}	y^{3+}	#2
1	114.09135	57.54931	38.70197	L				16
2	185.12847	93.06787	62.38101	A	1795.70277	898.35502	599.23911	15
3	314.17107	157.58917	105.39521	E	1724.66565	862.83646	575.56007	14
4	442.22965	221.61846	148.08140	Q	1595.62305	798.31516	532.54587	13
5	513.26677	257.13702	171.76044	A	1467.56447	734.28587	489.85968	12
6	642.30937	321.65832	214.77464	E	1396.52735	698.76731	466.18064	11
7	798.41049	399.70888	266.80835	R	1267.48475	634.24601	423.16644	10
8	961.47381	481.24054	321.16279	Y	1111.38363	556.19545	371.13273	9
9	1076.50076	538.75402	359.50510	D	948.32031	474.66379	316.77829	8
10	1191.52771	596.26749	397.84742	D	833.29336	417.15032	278.43597	7
11	1322.56821	661.78774	441.52759	M	718.26641	359.63684	240.09366	6
12	1393.60533	697.30630	465.20663	A	587.22591	294.11659	196.41349	5
13	1560.60369	780.80548	520.87275	S-Phospho	516.18879	258.59803	172.73445	4
14	1631.64081	816.32404	544.55179	A	349.19043	175.09885	117.06833	3
15	1762.68131	881.84429	588.23195	M	278.15331	139.58029	93.38929	2
16				K	147.11281	74.06004	49.70912	1

Supplementary Figure 15. (Related to Figure 5d) a. MS/MS spectra of the trypsin-digested YWHAH-derived peptides LAEQAERYDDMASAMK in control (MH+:1828.82279, upper panel) and phosphorylated (MH+: 1908.79209, lower panel). The coverage of YWHAH protein was 63% and 65% in control and phosphorylated group, respectively, where protein sequence coverage was calculated based on peptide sequence comparison by Proteome Discoverer (v1.2.0.208 with SEQUEST algorithm) (NCBI Reference Sequence: NP_003396.1, 14-3-3 protein eta [Homo sapience]) b. Fragment ions detected in MS/MS spectra of LAEQAERYDDMASAMK in un-phosphorylated. c. Fragment ions detected in MS/MS spectra of LAEQAERYDDMASAMK in phosphorylated. Matched b ions are colored in red and y ions in blue.

a

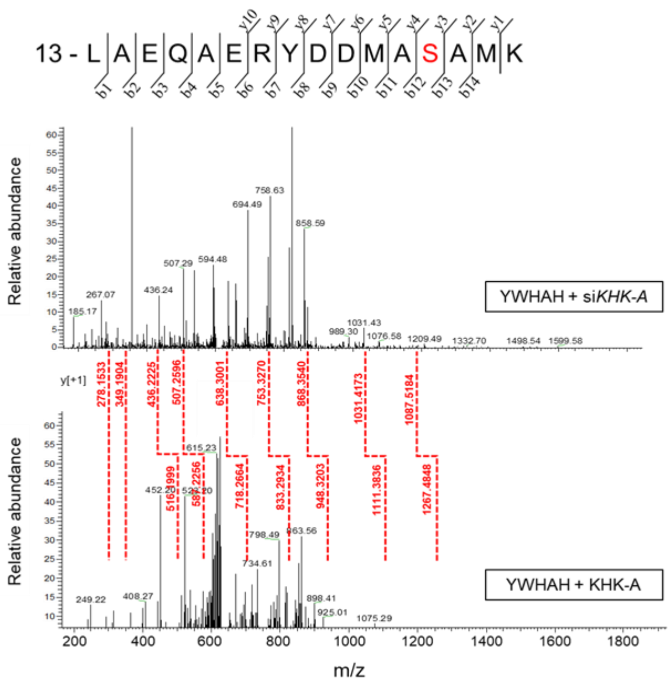

b

#1	b^{+}	b^{2+}	b^{3+}	Seq.	y^{+}	y^{2+}	y^{3+}	#2
1	114.09135	57.54931	38.70197	L				16
2	185.12847	93.06787	62.38101	A	1715.73644	858.37186	572.58366	15
3	314.17107	157.58917	105.39521	E	1644.69932	822.85330	548.90462	14
4	442.22965	221.61846	148.08140	Q	1515.65672	758.33200	505.89042	13
5	513.26677	257.13702	171.76044	A	1387.59814	694.30271	463.20423	12
6	642.30937	321.65832	214.77464	E	1316.56102	658.78415	439.52519	11
7	798.41049	399.70888	266.80835	R	1187.51842	594.26285	396.51099	10
8	961.47381	481.24054	321.16279	Y	1031.41730	516.21229	344.47728	9
9	1076.50076	538.75402	359.50510	D	868.35398	434.68063	290.12284	8
10	1191.52771	596.26749	397.84742	D	753.32703	377.16715	251.78053	7
11	1322.56821	661.78774	441.52759	M	638.30008	319.65368	213.43821	6
12	1393.60533	697.30630	465.20663	A	507.25958	254.13343	169.75804	5
13	1480.63736	740.82232	494.21730	S	436.22246	218.61487	146.07900	4
14	1551.67448	776.34088	517.89634	A	349.19043	175.09885	117.06833	3
15	1682.71498	841.86113	561.57651	M	278.15331	139.58029	93.38929	2
16				K	147.11281	74.06004	49.70912	1

c

#1	b^{+}	b^{2+}	b^{3+}	Seq.	y^{+}	y^{2+}	y^{3+}	#2
1	114.09135	57.54931	38.70197	L				16
2	185.12847	93.06787	62.38101	A	1795.70277	898.35502	599.23911	15
3	314.17107	157.58917	105.39521	E	1724.66565	862.83646	575.56007	14
4	442.22965	221.61846	148.08140	Q	1595.62305	798.31516	532.54587	13
5	513.26677	257.13702	171.76044	A	1467.56447	734.28587	489.85968	12
6	642.30937	321.65832	214.77464	E	1396.52735	698.76731	466.18064	11
7	798.41049	399.70888	266.80835	R	1267.48475	634.24601	423.16644	10
8	961.47381	481.24054	321.16279	Y	1111.38363	556.19545	371.13273	9
9	1076.50076	538.75402	359.50510	D	948.32031	474.66379	316.77829	8
10	1191.52771	596.26749	397.84742	D	833.29336	417.15032	278.43597	7
11	1322.56821	661.78774	441.52759	M	718.26641	359.63684	240.09366	6
12	1393.60533	697.30630	465.20663	A	587.22591	294.11659	196.41349	5
13	1560.60369	780.80548	520.87275	S-Phospho	516.18879	258.59803	172.73445	4
14	1631.64081	816.32404	544.55179	A	349.19043	175.09885	117.06833	3
15	1762.68131	881.84429	588.23195	M	278.15331	139.58029	93.38929	2
16				K	147.11281	74.06004	49.70912	1

Supplementary Figure 16. (Related to Supplementary Figure 9i) a. MS/MS spectra of the trypsin-digested YWHAH-derived peptides LAEQAERYDDMASAMK in control (MH+:1828.82151, upper panel) and phosphorylated (MH+: 1908.79428, lower panel). The coverage of YWHAH protein was 67% and 67% in control and phosphorylated group, respectively, where protein sequence coverage was calculated based on peptide sequence comparison by Proteome Discoverer (v1.2.0.208 with SEQUEST algorithm) (NCBI Reference Sequence: NP_003396.1, 14-3-3 protein eta [Homo sapience]). b. Fragment ions detected in MS/MS spectra of LAEQAERYDDMASAMK in un-phosphorylated. c. Fragment ions detected in MS/MS spectra of LAEQAERYDDMASAMK in phosphorylated. Matched b ions are colored in red and y ions in blue.

Supplementary Table 1. Nucleotide sequences of siRNAs

siRNA		Sequence
Pan	Control	5'- AUGAACGUGAAUUGCUCAATT -3'
human	KHK-A	5'- GUCAUCAUCAACGAGGCCAGUGGTA -3'
human	KHK-C	5'- CCAAUGGCAACCGUACCAUUGUGCT -3'
human	PRPS1	5'- CAAUGGAGAAUCCGUUUCUUACCTA -3'
human	ALDOB	5'- CUUGCUAGUCAUUGGAAUCAAGCCG -3'
human	ALOX12	5'- GAUCCAGUAUCACUUGCUGAACACT -3'
human	GLOD4	5'- GACUACAAGCUUGGCAAUGACUUTA -3'
human	YWHAH	5'- ACAGUGUGGUCGAAGCUUCUGAAGC -3'
human	YWHAH (3'UTR)	5'- GUUUUGGAAUUCAAUGGGUAAAUAA -3'
mouse	Ywhah	5'- CAAGAACUGCAAUGAUUUUCAGUAT -3'
mouse	Ywhah (3'UTR)	5'- GCAGUUUCAGAUAAACCUUCAUGGG -3'
human	LRRC59	5'- AAGCUAGACCUGAGUAAGAACAAGC -3'
human	KPNB1	5'- GAGGUGGCUUUACAAGGGAUAGAAT -3'
human	SNAI1	5'- CAACUGCAAAUACUGCAACAAGGAA -3'
mouse	Snai1	5'- ACAGUUUAUUGAUAUUCAAUAAAAT -3'
human	SNAI2	5'- ACUGAGUGACGCAAUCAAUGUUUAC -3'
mouse	Snai2	5'- UAUAUUUACUGACAGCUAGAUUGAA -3'

Supplementary Table 2. Nucleotide sequences of shRNAs

shRNA	Sequence
Control	5'- TTCTCCGAACGTGTCACGT -3'
Khk-a	5'- GGACTTACGATATGTGGTCCT -3'

Supplementary Table 3. Primers used in real-time quantitative PCR

Gene		Forward	Reverse
human	KHK-A_1	5'- TCATGGAAGAGAAGCAGATC -3'	5'- GGAGGTCATCCAGGACAAAA -3'
human	KHK-A_2	5'- TATTCTGTGGACCTACGCTA-3'	5'- CATAGTATAGGATGGTGCGG-3'
human	KHK-C_1	5'- TCATGGAAGAGAAGCAGATC -3'	5'- TGAAGTCGGCCACCAGGAAG -3'
human	KHK-C_2	5'- CATGTTGCTGACTTCCTGG-3'	5'- TTGGAGTTGTTGATGATGCA-3'
human	CDH1	5'- TCTGGATAGAGAACGCATTG -3'	5'- TGTTGTCATTCTGATCGGTT -3'
human	GAPDH	5'- GAGTCAACGGATTTGGTCGT -3'	5'- TTGATTTTGGAGGGATCTCG -3'
mouse	Cdh1	5’-AAGCAGCAATACATCCTTCA -3’	5- CTCTCGAGCGGTATAAGATG -3’
mouse	18S rRNA	5'- GTTAATTCCGATAACGAACG -3'	5'- CACAGACCTGTTATTGCTCA -3'

Supplementary Table 4. Clinical information on breast cancer patients

No.	Age	Sex	Organ	Diagnosis	LM	Histologic grade
1	59	F	Breast	Infiltrating duct carcinoma	0/19	II
2	48	F	Breast	Infiltrating duct carcinoma	0/16	II
3	42	F	Breast	Infiltrating duct carcinoma	5/19	II
4	37	F	Breast	Infiltrating duct carcinoma	8/8	III
5	37	F	Breast	Infiltrating duct carcinoma	0/20	III
6	55	F	Breast	Infiltrating duct carcinoma	0/19	II
7	55	F	Breast	Infiltrating duct carcinoma	20/20	II
8	36	F	Breast	Infiltrating duct carcinoma	0/14	II
9	52	F	Breast	Infiltrating duct carcinoma	5/24	III
10	40	F	Breast	Infiltrating duct carcinoma	0/11	II
11	51	F	Breast	Infiltrating duct carcinoma	0/1	III
12	55	F	Breast	Infiltrating duct carcinoma	0/19	III
13	60	F	Breast	Infiltrating duct carcinoma	0/16	III
14	45	F	Breast	Sarcomatoid carcinoma	3/23	III
15	38	F	Breast	Infiltrating duct carcinoma	3/13	III
16	53	F	Breast	Infiltrating duct carcinoma	3/14	II
17	48	F	Breast	Infiltrating duct carcinoma	2/15	II
18*	46	F	Breast	Intraductal papillary carcinoma	0/15	uk
19	40	F	Breast	Infiltrating duct carcinoma	0/10	III
20*	51	F	Breast	Atypical medullary carcinoma	1/16	uk
21	56	F	Breast	Infiltrating duct carcinoma	0/14	III
22*	45	F	Breast	Metaplstic carcinoma	0/17	uk
23	42	F	Breast	Infiltrating duct carcinoma	12/26	II
24	27	F	Breast	Infiltrating duct carcinoma	1/11	II
25	39	F	Breast	Infiltrating duct carcinoma	2/17	III
26	51	F	Breast	Infiltrating duct carcinoma	1/13	III
27	49	F	Breast	Infiltrating duct carcinoma	0/20	III
28	57	F	Breast	Infiltrating duct carcinoma	3/7	II
29	52	F	Breast	Infiltrating duct carcinoma	1/22	II
30	41	F	Breast	Infiltrating duct carcinoma	7/9	II
31	48	F	Breast	Infiltrating duct carcinoma	35/35	III
32	34	F	Breast	Infiltrating duct carcinoma	2/11	III
33*	37	F	Breast	Infiltrating duct carcinoma	22/23	III
34	58	F	Breast	Infiltrating duct carcinoma	19/22	III
35	37	F	Breast	Infiltrating duct carcinoma	17/19	III
36	66	F	Breast	Infiltrating duct carcinoma	4/11	III
37	51	F	Breast	Infiltrating duct carcinoma	3/16	III
38	41	F	Breast	Infiltrating duct carcinoma	15/21	III
39	56	F	Breast	Infiltrating duct carcinoma	5/13	II
40	47	F	Breast	Infiltrating duct carcinoma	11/13	II

LM, Lymph-node metastasis; Histologic grade, Nottingham Modification of Bloom-Richard system; uk, unknown; *, denotes data were exclusion from analysis.
