# Supplementary material for: Ketohexokinase-A acts as a nuclear protein kinase that mediates fructose-induced metastasis in breast cancer
Source: Nat Commun. 2020 Oct 28;11:5436. doi: 10.1038/s41467-020-19263-1 (PMC7595112; doi:10.1038/s41467-020-19263-1)
Supplement: Supplementary file 3 — Reporting Summary [file 41467_2020_19263_MOESM3_ESM.pdf]

nature research

Corresponding author(s): Jong-Wan Park
Last updated by author(s): Sep 22, 2020

Reporting Summary

Nature Research wishes to improve the reproducibility of the work that we publish. This form provides structure for consistency and transparency in reporting. For further information on Nature Research policies, see our Editorial Policies and the Editorial Policy Checklist.

Statistics

For all statistical analyses, confirm that the following items are present in the figure legend, table legend, main text, or Methods section.

n/a	Confirmed	
☐	☒	The exact sample size (*n*) for each experimental group/condition, given as a discrete number and unit of measurement
☐	☒	A statement on whether measurements were taken from distinct samples or whether the same sample was measured repeatedly
☐	☒	The statistical test(s) used AND whether they are one- or two-sided<br>*Only common tests should be described solely by name; describe more complex techniques in the Methods section.*
☒	☐	A description of all covariates tested
☒	☐	A description of any assumptions or corrections, such as tests of normality and adjustment for multiple comparisons
☐	☒	A full description of the statistical parameters including central tendency (e.g. means) or other basic estimates (e.g. regression coefficient) AND variation (e.g. standard deviation) or associated estimates of uncertainty (e.g. confidence intervals)
☐	☒	For null hypothesis testing, the test statistic (e.g. *F*, *t*, *r*) with confidence intervals, effect sizes, degrees of freedom and *P* value noted<br>*Give P values as exact values whenever suitable.*
☒	☐	For Bayesian analysis, information on the choice of priors and Markov chain Monte Carlo settings
☒	☐	For hierarchical and complex designs, identification of the appropriate level for tests and full reporting of outcomes
☐	☒	Estimates of effect sizes (e.g. Cohen's *d*, Pearson's *r*), indicating how they were calculated

Our web collection on statistics for biologists contains articles on many of the points above.

Software and code

Policy information about availability of computer code

Data collection	ImageJ 1.8.0<br>in vivo luminescence: live imaging 2.50.1<br>Confocal microscopy: Zeiss LSM 510<br>BioRad CFX manager v 3.1.1517.0823<br>Microsoft excel v 2013
Data analysis	GraphPad Prism v 5.0, and v 8.0. Used for all graphs and statistical analysis<br>Interactome analysis: Maxquant 1.5.5.1<br>PTM analysis: Proteome discoverer V1.2.0.208 with SEQUEST algorithm<br>ChromaTOF 5.40.12.0 used for quantification of fructose<br>in vivo luminescence: live imaging 2.50.1<br>Hex package v 8.0.0 for molecular docking analysis<br>GROMACS package (v 5.1.2) with CHARMM36 force field for NTP-ensemble MD simulating

For manuscripts utilizing custom algorithms or software that are central to the research but not yet described in published literature, software must be made available to editors and reviewers. We strongly encourage code deposition in a community repository (e.g. GitHub). See the Nature Research guidelines for submitting code & software for further information.

Data

Policy information about availability of data

All manuscripts must include a data availability statement. This statement should provide the following information, where applicable:

- Accession codes, unique identifiers, or web links for publicly available datasets
- A list of figures that have associated raw data
- A description of any restrictions on data availability

The mass spectrometry proteomics data have been deposited to the ProteomeXchange Consortium via the PRIDE partner repository with the dataset identifier PXD021035. The proteomics data referenced during the study are available in a public repository from the PRIDE website. All the other data supporting the findings of this study are available within the article and its supplementary information files. A reporting summary for this article is available as a supplementary Information file.

Field-specific reporting

Please select the one below that is the best fit for your research. If you are not sure, read the appropriate sections before making your selection.

☒ Life sciences ☐ Behavioural & social sciences ☐ Ecological, evolutionary & environmental sciences

For a reference copy of the document with all sections, see nature.com/documents/nr-reporting-summary-flat.pdf

Life sciences study design

All studies must disclose on these points even when the disclosure is negative.

Sample size	No statistical methods were used to predetermine sample size in all experiments with cell line(s). In case of mice experiments, sample size were determined based on published sample size usages for similar experiments, examples include (Cancer Res. 2018 Mar 1;78(5):1184-1199; Oncogene. 2018 Sep;37(38):5191-5204; Nat Commun. 2018 Oct 5;9(1):4113). Also the reason for choosing 7 mice per group was to use the minimum number of animals that can obtain statistically significant experimental results through non-parametric test.
Data exclusions	No data were excluded except for micro-tissue array analysis, the slide (Cat. No. CBA, Breast cancer-metastasis-normal) is consisting of n = 59 cancer tissue samples from 40 patients. 59 sample types are divided as follows: 40 samples from primary breast cancer; 10 samples from metastatic carcinoma; 9 samples from normal breast tissue. To analyze metastasis potential of KHK-A and YWHAH-pSer25 in breast cancer, we only analyzed 40 samples from primary breast cancer. During immunohistochemistry experiments, despite handled very carefully, 1 tissue sample was fall off repeatedly (sample # 33). The histologic grade was not defined in sample # 18, 20, 22. Therefore, sample #18, 20, 22, 33 were excluded in all related (Figure 10) analysis.
Replication	Experiments were performed multiple times. And most experiments were reproduced at least three times with similar results.
Randomization	For all animal experiments, mice were randomized prior to cancer cell injection. All other experiments were randomized using randomly assigned plates of cells per experiment.
Blinding	No aspects of the study were blinded. Except for animal studies. The investigators who performed animal studies were blinded for grouping.

Reporting for specific materials, systems and methods

We require information from authors about some types of materials, experimental systems and methods used in many studies. Here, indicate whether each material, system or method listed is relevant to your study. If you are not sure if a list item applies to your research, read the appropriate section before selecting a response.

Materials & experimental systems

n/a	Involved in the study	
☐	☒	Antibodies
☐	☒	Eukaryotic cell lines
☒	☐	Palaeontology and archaeology
☐	☒	Animals and other organisms
☒	☐	Human research participants
☒	☐	Clinical data
☒	☐	Dual use research of concern

Methods

n/a	Involved in the study	
☒	☐	ChIP-seq
☒	☐	Flow cytometry
☒	☐	MRI-based neuroimaging

Antibodies

Antibodies used	Slug (Santa Cruz Biotechnology, sc-166476(A-7) Mouse,1:K for WB; 1:50 for ChIP); N-cadherin (CDH2) (Santa Cruz Biotechnology, sc-7939(H-63), Rabbit 1:K for WB); Vimentin (Santa Cruz Biotechnology, sc-7558(S-20), Goat, 1:K for WB); ZEB-1(Santa Cruz

Biotechnology, sc-25388(H-102), Rabbit, 1:K for WB); β-tubulin(Santa Cruz Biotechnology, sc-9104(H-235), Rabbit, 1:5K for WB); GST-tag(Santa Cruz Biotechnology, sc-138(B-14), Mouse, 1:K for WB); PRPS1/2(Santa Cruz Biotechnology, sc100288(EE-17), Mouse, 1:K for WB, 1:100 for IP); Aldolase B (Santa Cruz Biotechnology, sc393278, C-11, Mouse, 1:K for WB); Lamin-B (Santa Cruz Biotechnology, sc-6216(C-20), Goat, 1:K for WB); Ketohexokinase(Santa Cruz Biotechnology, sc377411(B-6), Mouse, 1:500 for WB; 1:100 for IHC); KPNB1(Abcam, ab2811(3E9), Mouse, 1:K for WB); alpha-SMA (ACTA2)(Abcam, ab7817(1A4), Mouse, 1:K for WB); TWIST(Abcam, ab50581, Rabbit, 1:K for WB); SNAIL(Abcam, ab53519, Goat, 1:K for WB); YWHAH (CST, 9640, Rabbit, 1:K for WB; 1:100 for IHC; 1:50 for ChIP); Myc-tag(CST, 2278, Rabbit, 1:K for WB); GFP(CST, 2555, Rabbit, 1:K for WB; 1:100 for IF); SQSTM1/p62(CST, 5114T, Rabbit, 1:K for WB); phosphor-serine/threonine (ECM biosciences, PP2551, Rabbit, 1:K for WB); phosphor-tyrosine(ECM biosciences, PP2221, Rabbit, 1:500 for WB); LRRC59 (Novus biologicals, NBP1-93953, Rabbit, 1:K for WB; 1:200 for IF; 1:100 for IHC); 12-lipoxygenase (Novus biologicals, NBP1-90338, Rabbit, 1:K for WB); KHK-A (signalway antibody LCC, AB21708-2, Rabbit, 1:K for WB, 1:100 for IP, 1:50 for IHC); KHK-C (signalway antibody LCC, AB21709-2, Rabbit, 1:K for WB, 1:100 for IP, 1:50 for IHC); E-cadherin (CDH1)(Thermo Fisher Scientific, 131700, Mouse,1:K for WB; 1:50 for IHC); SQSTM1/p62 phospho-Ser28(Thermo Fisher Scientific, PA5-35409, Rabbit, 1:K for WB); GLOD4 (Genetex, GTX104484(N1C3), Rabbit, 1:K for WB); HIF-1alpha(Custom made,Biochem Biophys Res Commun. 2000 Feb 16;268(2):652-6. doi: 10.1006/bbrc.2000.2180 Rabbit, 1:K for WB); His(6)-tag(MBL, PM032, Mouse, 1:K for WB); FLAG(Sigma-Aldrich, F7425, Rabbit, 1:3k for WB; 1:200 for ChIP); HRP-conjugated rabbit anti-goat (Thermo Fisher Scientific, 81-1620,1:5K for WB); HRP-conjugated goat anti-rabbit (invtrogen, G21234, 1:5K for WB); HRP-conjugated goat anti-mouse (invtrogen, G21040, 1:5K for WB)

Validation

SLUG (Santa Cruz Biotechnology, sc-166476(A-7): WB, IP, IHC, IF; 77 citations; Santa Cruz biotechnology website antibody validation: 1) Western blot analysis of SLUG expression in HeLa, PC-3, MDA-MB-231, HepG2, RAW 264.7, SJRH30, hSLUG transfected 293 lysate, Whole cell lysate and mouse placenta tissue extract show SLUG band between 34, and 43 kDa. 2) Immunoperoxidase staining of formalin fixed, paraffin-embedded human breast tissue showing nuclear staining of SLUG

N-cadherin (CDH2) (Santa Cruz Biotechnology, sc-7939(H-63): WB, IP, IF, ELISA; 99 citations; Santa Cruz website antibody validation: 1) Western blot analysis of N-cadherin expression in mouse brain tissue extract shows N-Cadherin band at expected Mw at 132 kDa. 2) IF staining of normal mouse heart frozen section shows N-cadherin level. 3) IHC analysis – the expression level of N-Cadherin in human heart muscle tissue.

Vimentin (Santa Cruz Biotechnology, sc-7558(S-20): WB, IP, IF, Flow cytometry, ELISA; 57 citations; Santa Cruz biotechnology website antibody validation: 1) Western blot analysis of Vimentin expression in HISM whole cell lysate shows Vimentin band at expected Mw at 55 kDa.

ZEB-1 (Santa Cruz Biotechnology, sc-25388(H-102): WB, IP, IF, ELISA; 38 citations; Santa Cruz biotechnology website antibody validation does not provide since the production of antibody discontinued; Western blot validation using sc-25338: PMID: 17023432
Beta-tubulin (Santa Cruz Biotechnology, sc-9104(H-235): WB, IP, IHC; 317 citations; Santa Cruz biotechnology website antibody validation: 1) Western blot analysis of β2C Tubulin expression in non-transfected 293T: (sc117752), mouse β2C Tubulin transfected 293T: sc-126330, and K-562 whole cell lysates show beta-tubulin band between 43, and 69 kDa. 2) IF analysis of HeLa cells showing cytoplasmic localization of beta-tubulin. 3) IHC analysis of human breast tissue showing cytoplasmic staining of beta-tubulin.

GST-tag (Santa Cruz Biotechnology, sc-138(B-14): WB, IP; 1083 citations; Santa Cruz biotechnology website antibody validation: 1) Direct western blot analysis of Schistosoma japonicum recombinant GST fusion protein 2) Direct near-infrared western blot analysis of GST expression in Stat4 human recombinant, and Schistosoma japonicum recombinant. 3) Western blot analysis of human recombinant NFκB p50 fusion protein. 4) Western blot analysis of GST-tagged fusion proteins showing C-terminal GST-tagged Max, and N-terminal GST-tagged Bcl-6.

PRPS1/2 (Santa Cruz Biotechnology, sc100822(EE-17): WB, IP; 2 citations; Santa Cruz biotechnology website antibody validation: 1) Western blot analysis of PRPS1/2 expression in HeLa whole cell lysate shows PRPS1/2 band between 25, and 37 kDa.

Aldolase B (Santa Cruz Biotechnology, sc393278 (C-11): WB, IP, IHC, IF; 1 citation; Santa Cruz biotechnology website antibody validation: 1) Western blot analysis of Aldolase B expression in non-transfected: sc-117752, and mouse Aldolase B transfected: sc-124950, also in 293T whole cell lysates and mouse liver, and human liver tissue extracts shows Aldolase B band between 34, and 50 kDa. 2) IHC analysis of mouse liver tissue showing cytoplasmic level of aldolase B.

Lamin-B (Santa Cruz Biotechnology, sc-6216(C-20); WB, IP, IF, IHC; 209 citations; Santa Cruz biotechnology website antibody validation: 1) Western blot analysis of Lamin B expression in CCRF-CEM cell lysate show Lamin-B band between 55, and 90 kDa. 2) IHC analysis of human breast tumor showing Lamin B localization (nuclear envelope) 3) IF analysis of F9 cells showing nuclear lamina localization using sc-6216.

Ketohexokinase (Santa Cruz Biotechnology, sc377411(B-6): WB, IP IF, IHC; 3 citations; Santa Cruz biotechnology website antibody validation: 1) Western blot analysis of Ketohexokinase expression in HeLa, RAW 264.7, KNRK, and PC-12 cell lysates show Ketohexokinase band between 23, and 34 kDa. 2) IHC analysis of human liver tissue showing cytoplasmic, membrane and nuclear staining level of ketohexokinase in liver. 3) IF analysis of HeLa cells showing cytoplasmic localization of ketohexokinase.

alpha-SMA (ACTA2) (Abcam, ab7817(1A4): WB, IF, IHC, Flow Cyt; 498 citations; Abcam website antibody validations: 1) Western blot analysis of HeLa whole cell lysate, and ACTA2 knock out Hela Cell lysate, and HEK-293 whole cell lysate shows alpha-SMA band between 37, and 50 kDa. 2) IHC – the expression level of alpha-SMA in human breast ductal carcinoma tissue section, performed on a Leica Bond™ system using the standard protocol.

TWIST (Abcam, ab50581): IF; 99 citations; Abcam website antibody validation: 1) IF – the expression level of TWIST in human glioblastoma and human melamona Mel15 cells; Western blotting validation: PMID: 32323782, PMID: 31004656

SNAIL (Abcam, ab53519): WB, IHC, 128 citations; Abcam website antibody validation: 1) Western blot analysis of Rat kidney lysate show SNAIL band between 25, and 37 kDa. 2) IHC analysis of mouse tissue shows the expression of SNAIL.

YWHAH (CST, 9640): WB, IP; 5 citations; CST website antibody validation: 1) Western blot analysis of extracts from HeLa, NIH/3T3,

PC12 and COS cells show YWHAH band between 20, and 30 kDa.

Myc-tag (CST, 2278): WB, IP, IF, Flow Cyt; 308 citations; CST website antibody validation: 1) Western blot analysis performed using extracts from untransfected control cells, and transfected cells overexpressing Myc-Bcl-2, using Bcl-2 Antibody #2872 and Myc-Tag (71D10). 2) IF analysis of 293 cells stably expressing Myc-tagged ADORA2A versus wild-type 293 cells using CST, 2278.

GFP (CST, 2555): WB, IHC; 135 citations; CST website antibody validation: 1) Western blot analysis of extracts from COS-7 cells, control or expressing GFP-tagged fusion protein. 2) Immunohistochemical analysis of paraffin-embedded HCC827 cells control or GFP-transfected using GFP Antibody; IF analysis validation: PMID: 32404436.

SQSTM1/p62(CST, 5114T): WB; 572 citations; CST website antibody validation: 1) Western blot analysis of extracts from HeLa, Jurkat, THP-1, A20, YB2/0 cell lysate show one or double band of SQSTM1/p62 at 60 kDa.

phosphor-serine/threonine (ECM biosciences, PP2551): WB, IP, IF, ELISA; 11 citations; ECM biosciences website validation: 1) Western blot analysis of A431 cells treated with calyculin A (100 nM) for 30 min then treated with lambda phosphatase. The blot was probed with anti-Phosphoserine/threonine rabbit polyclonal at 1:1000. This antibody was cross-adsorbed to unphosphorylated peptide then affinity purified using a mix of phosphoserine and phosphothreonine peptides.

phosphor-tyrosine(ECM biosciences, PP2221): WB, IP, IF, ELISA; 3 citations; ECM biosciences website validation: 1) Immunofluorescence analysis: labeling of phosphotyrosine in control and pervanadate-treated A431 cells; Western blot validation: PMID: 25825764, PMID: 23108973.

LRRC59 (Novus biologicals, NBP1-93953): WB, IF, IHC; No citation was reported; Novus biologicals website validation: 1) Western Blot Analysis in mouse cell line NIH-3T3, rat cell line NBT-II, and human CACO-2 lysate show LRRC59 band at 35 kDa. 2) IF analysis of human cell line A-431 shows localization of LRRC59 (endoplasmic reticulum) 3) IHC analysis of human cerebral cortex, colon, liver and lymph node using Anti-LRRC59 antibody(NBP1-93953).

12-lipoxygenase (Novus biologicals, NBP1-90338): WB, IHC; No citation was reported; Novus biologicals website validation: 1) Western Blot: Analysis in human cell line HELA. 12-Lipoxygenase band show at 70 kDa. 2) IHC: Staining of human heart muscle with NBP1-90338 shows distinct positivity in myocytes.

KHK-A (signalway antibody LCC, AB21708-2): WB, IHC; 2 citations; WB, and IHC validation: PMID: 27088854; PMID: 31032410.

KHK-C (signalway antibody LCC, AB21709-2): WB, IHC; 2 citations; WB and IHC validation: PMID: 27088854; PMID: 31032410.

E-cadherin (CDH1)(Thermo Fisher Scientific, 131700): WB, IP, IHC, IF, IA, Flow Cyt, ELISA; 259 citations; Thermofisher website validation: 1) Western blot analysis in MCF-7, T-47D, MDA-MB-231, SW480, Caco-2, and BJ lysate show E-Cadherin band at 110 kDa.

SQSTM1/p62 phospho-Ser28(Thermo Fisher Scientific, PA5-35409): WB; 1 Citation: PMID: 31032410.

GLOD4 (Genetex, GTX104484(N1C3)): WB, IHC; No citation was reported; Genetex website validation: 1) Western blot analysis in HepG2, and Mouse brain lysate show GLOD4 band nearby 34 kDa. 2) IHC analysis show GLOD expression level in human gastric tissue.

HIF-1alpha(Reference PMID: 10679259, Rabbit): WB; Selected cited papers: PMID: 15919761, PMID: 11834720, PMID: 30837551, PMID: 31332228, PMID: 30659265

His(6)-tag(MBL, PM032): WB, IP; 9 citations; MBL website validation: 1) Western blot analysis of His-tagged recombinant proteins (https://www.mblintl.com/products/pm032/)

FLAG (Sigma-Aldrich, F7425): WB, IP, IF; 1791 citations; Sigmaaldrich website validation: 1) Western blot analysis using FLAG-tagged recombinant proteins

HRP-conjugated rabbit anti-goat (Thermo Fisher Scientific, 81-1620): WB, IHC, IF; 16 citations; WB validation: PMID: 29777158, PMID: 28056338, PMID: 27619946

HRP-conjugated goat anti-rabbit (invtrogen, G21234): WB, IP, IHC, ELISA; 182 citations; Thermofisher website validation: 1) Western blot analysis was performed on whole cell lysate of Rat Brain (Lane 1), SH-SY5Y (Lane 2), U-87 MG (Lane 3), HEL 92.1.7 (Lane 4) and Hep G2 (Lane 5), MCF-7, A375, Jurkat, Mouse small intestine, PANC-1, THP-1, and HeLa. Primary: Anti-Cyclophilin D Rabbit Polyclonal Antibody (Product # PA3-023) and detected by chemiluminescence using Goat anti-Rabbit IgG (H+L) Secondary Antibody, HRP conjugate (G21234)

HRP-conjugated goat anti-mouse (invtrogen, G21040): WB, IHC, ELISA; 227 citations; Thermofisher website validation: 1) Western blot analysis was performed on whole cell extracts (30 µg lysate) of K-562 and U-87 MG. Primary: SOD2 Mouse Monoclonal Antibody (Product# MA1-106)and detected by chemiluminescence using Goat anti-Mouse IgG (H+L) Cross-Adsorbed Secondary Antibod, HRP conjugated. (G21040).

For S25-phosphorylated YWHAH, the antibody was raised using a phage display technology through a commercial facility (Bioneer, Daejoen, South Korea). Positive clone was chosen by ELISA screening. We submitted the relevant documents with reporting summary (1. the experimental validation sheet, 2. data sheet, and 3. certificates of synthesized peptides (YWHAH, and YWHAH-pSer25).

Eukaryotic cell lines

Policy information about cell lines

Cell line source(s)	Human breast cancer (MDA-MB-231 and MCF-7), embryonic kidney (HEK293T), glioblastoma (U87 and U251), lung adenocarcinoma (H1299 and A549), colon cancer (HCT116, and DLD-1), renal cancer (786O and RCC4), cervical cancer (SiHa and HeLa), ovarian cancer (SKOV3), bone cancer (U2OS), hepatocellular carcinoma (HepG2, and Hep3B) cell lines were obtained from American Type Culture Collection (ATCC; Manassas, VA); human pancreatic cancer (MIApaca2 and PanC1), human prostate cancer (PC3 and DU145) and mouse breast cancer (MTV-TM-011) cell lines from Korean Cell Line Bank (Seoul, Korea)
Authentication	HepG2, A549, RCC4, H1299, MDA-MB-231, 786O, U87, U251, SiHa, and HeLa were genetically authenticated by Korea cell line bank (The Fingerprinting of cell lines by AmplFLSTR identifier PCR amplification kit was tested. STR Kit: AmpLTLSTR identifier PCR amplification kit (Applied Biosystems, Foster, CA, cat. 4322288; Analysis Methods : 3730 DNA analyzer (Applied Biosystems, Foster, CA); GeneMapper ID v 3.2 (Applied Biosystems, Foster, CA). The rest of cell lines (MCF-7, HEK293T, DLD-1, SKOV3, U2OS, Hep3B, PC3, DU145, and MTV-TM-011) used in the study have not been authenticated.
Mycoplasma contamination	All used cell lines in this study were tested negative to mycoplasma by using MycoAlert mycoplasma detection kit (LT07-417, Lonza)
Commonly misidentified lines (See ICLAC register)	No misidentified cell lines were used in this study.

Animals and other organisms

Policy information about studies involving animals; ARRIVE guidelines recommended for reporting animal research

Laboratory animals	6-7-week-old female balb-c/nude mice (OrientBio, Seongnam, South Korea) were used for breast cancer xenograft. All mice housed in a specific pathogen-free room under temperature (23℃± 3℃),and humidity (40~60%) controlled conditions with 12/12h light/ dark cycle. Also the Institute for experimental animal center have a system that can ventilate 15 - 16 times/ hour with an airflow rate of 13 - 18 cm/ sec.
Wild animals	No wild animals were used in the study.
Field-collected samples	No field collected samples were used in the study.
Ethics oversight	All animal experiments were carried out with an approved protocol proposal from the Seoul National University Institutional Animal Care and Use Committee (Seoul, Korea), Approval No. SNU-170721-1-5 for Figure 2; SNU-190712-2-1 for Supplementary Figure 6; SNU-190819-1 for Figure 6.

Note that full information on the approval of the study protocol must also be provided in the manuscript.
